# bHLH Transcription Factor NtMYC2a Regulates Carbohydrate Metabolism during the Pollen Development of Tobacco (*Nicotiana tabacum* L. cv. TN90)

**DOI:** 10.3390/plants11010017

**Published:** 2021-12-22

**Authors:** Shiquan Bian, Tian Tian, Yongqiang Ding, Ning Yan, Chunkai Wang, Ning Fang, Yanhua Liu, Zhongfeng Zhang, Hongbo Zhang

**Affiliations:** Tobacco Research Institute of Chinese Academy of Agricultural Sciences, Qingdao 266101, China; bsq20200130@163.com (S.B.); tiantian01@caas.cn (T.T.); dyq910420@163.com (Y.D.); yanning@caas.cn (N.Y.); wangchunkai1990@outlook.com (C.W.); fangning010@163.com (N.F.); liuyanhua@caas.cn (Y.L.); zhangzhongfeng@caas.cn (Z.Z.)

**Keywords:** NtMYC2a, carbohydrate metabolism, starch, pollen maturation, tobacco

## Abstract

Basic helix-loop-helix (bHLH) transcription factor MYC2 regulates plant growth and development in many aspects through the jasmonic acid (JA) signaling pathway, while the role of MYC2 in plant carbohydrate metabolism has not been reported. Here, we generated *NtMYC2a*-overexpressing (*NtMYC2a*-OE) and RNA-interference-mediated knockdown (*NtMYC2a*-RI) transgenic plants of tobacco (*Nicotiana tabacum* L. cv. TN90) to investigate the role of NtMYC2a in carbohydrate metabolism and pollen development. Results showed that NtMYC2a regulates the starch accumulation and the starch-sugar conversion of floral organs, especially in pollen. The RT-qPCR analysis showed that the expression of starch-metabolic-related genes, *AGPs*, *SS2* and *BAM1,* were regulated by NtMYC2a in the pollen grain, anther wall and ovary of tobacco plants. The process of pollen maturation was accelerated in *NtMYC2a*-OE plants and was delayed in *NtMYC2a*-RI plants, but the manipulation of *NtMYC2a* expression did not abolish the pollen fertility of the transgenic plants. Intriguingly, overexpression of *NtMYC2a* also enhanced the soluble carbohydrate accumulation in tobacco ovaries. Overall, our results demonstrated that the bHLH transcription factor NtMYC2a plays an important role in regulating the carbohydrate metabolism during pollen maturation in tobacco.

## 1. Introduction

In flowering plants, pollen development is a complex and important physiological process which determines pollination, fertilization and hybrid seed production [1,2,3,4]. The pollen grains of angiosperms are derived from the microspore mother cells (also called pollen mother cells) in the pollen sac. This process involves multiple primary and secondary metabolism, among which the carbohydrate metabolism plays an important role [1,5,6]. As the richest storage carbohydrate in plants, starch provides energy for growth and development. Unusual starch accumulation in the tapetum cells results in pollen abortion during the late stage of anther and pollen development [7,8,9]. The phosphorylation and degradation of starch in pollen also affects its maturation [10]. Recent study also revealed that male sterility may result from the disorder of starch synthesis and utilization in pollen grains [11].

Starch metabolism is a complex biochemical process which requires the participation of various enzymes. ADP-glucose pyrophosphorylase (AGPase) controls the conversion of carbon to starch in chloroplast, and a deficiency of AGPase leads to pollen sterility [12,13,14]. In addition, starch synthases (SSs) [15], branching enzymes (BEs) [16] and debranching enzymes (DBEs) [17] are also involved in amylopectin synthesis. The complete degradation of starch is achieved by endo-acting α-amylases (AMYs) [18], exo-acting β-amylases (BAMs) [19] and α-glucan debranching enzymes (DBEs) (ISA and LDA) [20,21]. In plants, starch and sugar can be converted into each other at different developmental stages. Specific di- and oligosaccharides have been shown to regulate the pollen germination [22]. Moreover, the perturbation of sugar partitioning and signaling could lead to male sterility, indicating that sugar in the anther also plays an important role in pollen development [23,24].

Hormones are reported to influence plant development by regulating carbohydrate metabolism. For example, auxin influences pollen development by regulating the sugar signaling pathway [25]. Plant hormone jasmonate (JA), which plays an important role in improving the defense ability of plants against abiotic and biotic stresses, has also been proved to participate in regulating filament elongation, anther dehiscence and pollen development [26,27,28,29]. In the JA signaling pathway, the basic helix-loop-helix (bHLH) transcription factor MYC2 acts as the master [30,31]. Arabidopsis MYC2/3/4/5 interacts with the R2R3-MYB transcription factors MYB21 and MYB24 to regulate anther development and seed production through the JA signaling pathway [32]. MYC2/3/4/5 functions redundantly to regulate JA-induced root growth inhibition and plant defense against insect attack and pathogen infection [33]. Recent study also revealed the crucial role of MYC2/3/4 in wound-induced JA accumulation by regulating the transcription of genes involved in JA biosynthesis and catabolism [34]. Moreover, MYC2 plays a role in flavonoid biosynthesis in JA-treated Arabidopsis and specifically recognizes and binds to the G-box motif of the putrescine N-methyltransferase (PMT) promoter to regulate JA-induced nicotine biosynthesis in tobacco [35,36]. In addition, three bHLH-type transcription factors, ABA-INDUCIBLE BHLH-TYPE TRANSCRIPTION FACTOR/JA-ASSOCIATED MYC2-LIKE1 (JAM1) [37], and JA-ASSOCIATED MYC2-LIKE2 and JAM3 have been reported to negatively regulate JA-mediated male fertility [38]. Studies in tobacco showed that NtMYB305, the tobacco homologue of Arabidopsis MYB21, is a regulator of starch metabolism in the pollen grains, anther wall and floral nectary [39], however, the function of MYC2 in the carbohydrate metabolism during pollen development still remains unclear.

In this study, we developed the *NtMYC2a* RNA interference (*NtMYC2a*-RI) and the *NtMYC2a*-overexpressing (*NtMYC2a*-OE) transgenic plants and examined the starch and sugar contents of the pollen grains, anther walls and ovaries in different stages of these transgenic plants. Results showed that NtMYC2a regulates the starch metabolism in the pollen grains, anther walls and ovaries and plays a role in the process of pollen maturation.

## 2. Materials and Methods

### 2.1. Plant Materials and Growth Conditions

Tobacco (*N. tabacum* L.) cultivar TN90 was used as the parental plant for developing the transgenic plants in this study. All plants were cultivated in an indoor growth room at 23 °C with a photoperiod of 14hlight/10hdark.

### 2.2. Characterization of the Flower Development Stages

The development of tobacco flowers is divided into 14 stages in this study according to previous studies [39]. The characteristics of these floral development stages are as follows: S4: the sepals are fully expanded, the corolla is at the tip of the sepals and the tapetum is beginning to degenerate; S6: the corolla tube begins to appear at the tip of the calyx; S9: corolla tube enlarges gradually, petal tip color is pink, ovary milky yellow, flower nectary light orange; S12: deep pink flowers open and release pollen, ovaries milky yellow, flower nectaries bright orange with abundant nectar; S14: the corolla is dark purple and begins to shrink, the nectar is gone and the ovary is green and full.

### 2.3. Vectors Construction and Generation of Transgenic Plants

To generate *NtMYC2a*-OE transgenic plants, the full-length coding region of *NtMYC2a* was amplified from tobacco cv. TN90 plants and inserted into the pENTR-D-TOPO^®^ vector (Invitrogen, Waltham, MA, USA) at the *Not* I and *Asc* I restriction sites and, finally, integrated into pBin-attR-HA vector to produce the pBin-*NtMYC2a*-HA vector for expressing HA (hemagglutinin)-tagged NtMYC2a in plants by LR recombination with Gateway^®^ LR Clonase™ (Thermo Fisher Scientific, Waltham, MA, USA), according to the instructions of manufacturer. The resultant vector pBin-*NtMYC2a*-HA and the vector pBin-*NtMYC2aΔ*-RNAi, described in our previous study [36], were introduced into *Agrobacterium tumefaciens* LBA4404 and transformed into tobacco by the leaf-disk transformation method, respectively [39]. The plants transformed with the empty vector (vector pBin-HA or pBin-RNAi) were used as control. The T-region of these vectors carries an *NPTII* gene to provide kanamycin-resistance for transgenic plants. Three independent transgenic lines for each type of transgene were used in all the assays. All primers used for vector construction are listed in Supplemental Table S1. The vector sequences are shown as Supplemental Figures S1 and S2.

### 2.4. RT-qPCR

Total RNA was extracted using the Total RNA Extraction Reagent (Vazyme, Nanjing, Jiangsu, China) according to the manufacturer’s instructions. First-strand cDNA samples were synthesized by reverse transcription using a HiScript III All-in-one RT SuperMix Perfect for qPCR kit (Vazyme, Nanjing, Jiangsu, China) and were used as templates for RT-qPCR. Reactions were performed using a QuantStudioTM 5 Real-Time PCR System (Thermo Fisher Scientific, Waltham, MA, USA) with ChamQ Universal SYBR qPCR Master Mix (Vazyme, Nanjing, Jiangsu, China). For RT-qPCR analysis, three biological replications were used to calculate the relative expression value. *NtActin* expression was used as an internal control. Primers for RT-qPCR are shown in Supplemental Table S1.

### 2.5. Western Blotting

Tobacco leaves (about 0.1 g) were collected and ground in liquid nitrogen. The total protein was extracted with 200 μL 2×loading buffer (0.125 M Tris-HCl, pH 6.8, 25% glycerin, 5% SDS, 2.5% β-mercaptoethanol, 0.1% bromophenol blue) and separated by a 12% SDS-polyacrylamide gel and transferred to the PVDF membrane. The block was performed using 5% skim milk at 4 °C overnight. For the detection of NtMYC2a protein, the anti-HA primary antibody (Roche, Basel, Switzerland) and the HRP-labeled goat anti-mouse secondary antibody (Affinity Biosciences, Cincinnati, OH, USA) were used in turn and the signal was captured by fluorescence image analysis system (Tanon 5200, Shanghai, China) with the detection substrate (Pierce^®^ ECL Western Blotting Substrate, Thermo Scientific, Waltham, MA, USA). As the control, NtActin was detected by mouse anti-actin primary antibody (CoWin Biosciences, Taizhou, Jiangsu, China) and the above-described secondary antibody using the same visualization method.

### 2.6. Histological Assay

In order to observe the development of anther and ovary, the anthers and ovaries at different stages were vacuum fixed in FAA fixation solution (10% formaldehyde, 50% alcohol and 5% acetic acid) at room temperature and dehydrated in a graded ethanol series, embedded with Technovit^®^7100 (Hanau, Hesse-Darmstadt, Germany) plastic embedding kit, sectioned to 1.5–2 μm and then dried at 40 °C. The sections of anther were stained in I_2_/KI solution for 3 min, and the sections of ovary were stained in toluidine blue solution for 5 min, rinsed briefly with water 3 times and then photographed using a transmitted light microscope.

### 2.7. Pollen Germination Assay

Modified method of Arun (2011) was carried out in the pollen germination assays in this study. The improved germination medium contained 15% (*w*/*v*) sucrose, 0.01% (*w*/*v*) boric acid, 5 mM Ca(NO_3_)_2_, 1 mM MgSO_4_, 5 mM CaCl_2_ at pH 7.5 and 1.5% (*w*/*v*) agar. The pollen grains of mature anthers were released onto the medium directly and incubated in the dark at 23 °C for 10 h. The germination was observed and photographed under a stereoscopic microscope.

### 2.8. Starch and Sugar Analyses

The starch qualitative detection of tobacco pollen grain was performed using I_2_/KI solution (1% (*w*/*v*) I_2_ in 3% (*w*/*v*) KI) at S4, S6 and S9. Pollen grains were stained by I_2_/KI solution for 10 min and photographed using a stereoscopic microscope. For starch quantitative detection of indicated organs, a Starch Assay Kit (Solarbio, Beijing, China) was used according to the manufacturer’s instructions. Five ovaries, anther wall from 30 anthers and pollen grains from 30 anthers were collected as a single sample and measured respectively. Values were measured based on three replicates for each plant line; the value for wide-type plants was the average of three lines, and that for the *NtMYC2a*-OE and *NtMYC2a*-RI plants was the average of five lines. The total soluble sugar in the anther wall was measured using the Total Soluble Sugar Assay Kit (Solarbio, Beijing, China) according to the manufacturer’s instructions.

### 2.9. Statistical Analysis

Statistical analyses of quantitative data were performed using Microsoft Excel. Statistical significance was assessed by comparison with the control (wild-type or empty-vector transformed plants) using one-way analysis of variance followed by a two-tailed Student’s *t*-test: * *p* < 0.05; ** *p* < 0.01.

### 2.10. Accession Numbers

The Tobacco Genome Initiative numbers for genes mentioned in this article are as follows: *NtMYC2a* (LOC107820916), *NtAGPs* (AB018190), *NtSS2* (JX264160), *NtBAM1* (KF420482) and *NtActin* (X63603).

## 3. Results

### 3.1. Generation of NtMYC2a Transgenic Tobacco Lines

**Figure 1:**
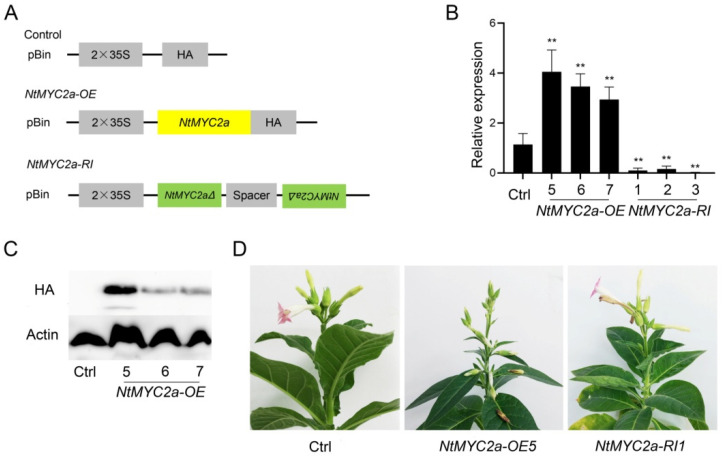
Identification of *NtMYC2a* overexpression and RNAi transgenic plants. (**A**) Schematic diagrams of the constructions used in this study. *NtMYC2a* in yellow box represents the full-length coding region of *NtMYC2a*, and the *NtMYC2a∆* in green box represents the selected *NtMYC2a* fragment for RNA interference; (**B**) Relative expression levels of *NtMYC2a* in *NtMYC2a*-OE and *NtMYC2a*-RNAi plants. The third leaves of 8-week-old *NtMYC2a*-OE, *NtMYC2a*-RNAi and the corresponding control plant were used in the RT-qPCR assays. Three biological replicates and two technical repeats were performed. Data are shown as means ± SD. ** *p* < 0.01 (Student’s *t*-test); (**C**) Detection of expressed HA-tagged NtMYC2a protein in control and *NtMYC2a*-OE plants by Western blot. Anti-HA and anti-actin antibodies were used in the blotting, respectively; (**D**) Phenotypes of control, *NtMYC2a*-OE and *NtMYC2a*-RI plants at flowering stage.

The full-length coding region of *NtMYC2a* was amplified and fused to the pBin-attR-HA vector containing constitutive 2×CaMV 35S promoter to generate *NtMYC2a*-overexpressing (*NtMYC2a*-OE) tobacco lines (Figure 1A). Meanwhile, a 451 bp fragment of *NtMYC2a* (*NtMYC2aΔ*), which was employed in our previously study [37], was used to develop *NtMYC2a*-RNA-interference (*NtMYC2a*-RI) lines (Figure 1A). Results of RT-qPCR analysis showed that the transcriptions of *NtMYC2a* increased in *NtMYC2a*-OE5/6/7 transgenic lines and decreased in *NtMYC2a*-RI1/2/3 lines (Figure 1B). Additionally, as shown in Figure 1C, the *NtMYC2a*-HA proteins were detected in lines *NtMYC2a*-OE5/6/7. These suggest that the expression of *NtMYC2a* was effectively up-regulated in *NtMYC2a*-OE plants and down-regulated in *NtMYC2a*-RI plants. Interestingly, we observed that overexpression of *NtMYC2a* leads to premature flower shedding, suggesting a role of NtMYC2a in flower development in tobacco (Figure 1D).

### 3.2. NtMYC2a Regulates Starch Accumulation in Pollen

**Figure 2:**
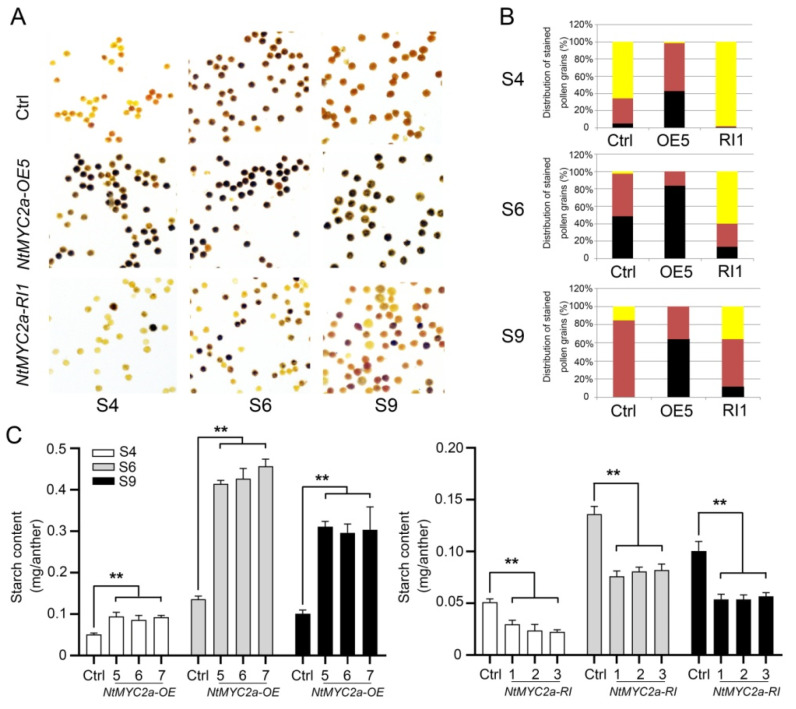
NtMYC2a regulates starch accumulation of pollen. (**A**) Pollen grains of *NtMYC2a*-OE, *NtMYC2a*-RI and control plants at S4, S6 and S9 stained by I_2_/KI solution; (**B**) Distribution of stained pollen grains in *NtMYC2a*-OE, *NtMYC2a*-RI and control plants at S4, S6 and S9. The distribution of stained pollen grains is shown as a percentage of total grains (5000 grains) counted for each of the iodine staining intensity categories. The black, brown and yellow boxes correspond to dark-stained, light-stained and unstained pollen grains, respectively. For each stage, five flowers were sampled from five different plants, and 1000 pollen grains were counted in each flower. In total, 5000 pollen grains were counted at each stage, giving standard error values that are very small and, thus, are not shown here; (**C**) Analysis of starch content in pollen grains at indicated floral development stages. Data are shown as means ± SD. Significant differences from the data of control plants are indicated: ** *p* < 0.01 (Student’s *t*-test).

In order to disclose the function of NtMYC2a on starch accumulation during pollen development, pollen grains of *NtMYC2a*-OE, *NtMYC2a*-RI and control plants were stained by iodine at the S4, S6 and S9 stages, respectively. Results showed that the pollen grains of control plants were stained yellow to brown at S4 and were stained dark brown to black due to the accumulation of amylose (Figure 2A,B). Compared with the control plants, the rate of black-stained pollen grains in the *NtMYC2a*-OE plants reached over 40% earlier at the S4 stage, and 80% pollen grains of *NtMYC2a*-OE plants stained black at S6 stage (Figure 2A,B). Most of the pollen grains of the *NtMYC2a*-RI plants could not be stained by iodine at the S4 stage, and only about 13.5% of them could be stained dark at the S6 stage (Figure 2A,B). At the S9 stage, the pollen grains of the control plants were stained brown due to the conversion of amylose to amylopectin; those of the *NtMYC2a*-OE plants were stained black due to the over-accumulation of amylose, while those of the *NtMYC2a*-RI plants had over 50% stained yellow and the rest stained brown to black (Figure 2A,B). These findings suggest that the starch accumulation in pollen grains was enhanced by overexpression of *NtMYC2a* and was delayed by the silencing of *NtMYC2a*.

Furthermore, the starch content in pollen grains of the transgenic and control plants at the indicated floral development stages was quantified. Compared with the control plants, the starch content of pollen grains from S4 to S9 significantly increased in *NtMYC2a*-OE plants, while decreased by nearly 50% in *NtMYC2a*-RI plants (Figure 2C). This indicates that NtMYC2a plays a positive role in starch accumulation during pollen development.

### 3.3. Starch Metabolism in Anther Wall and Ovary Are Also Regulated by NtMYC2a

**Figure 3:**
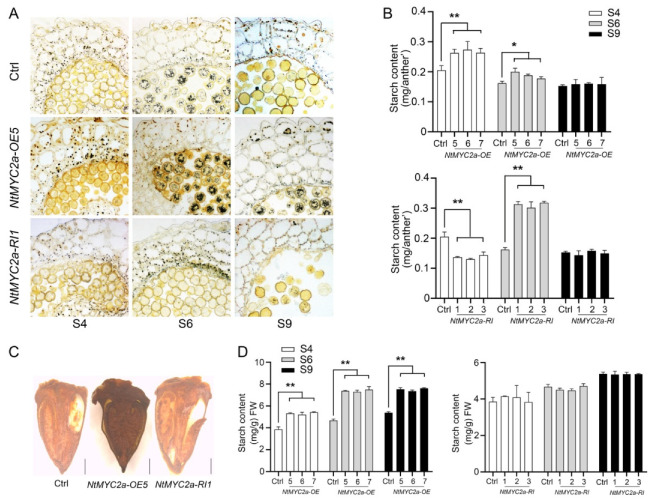
Total analysis of starch content in anther wall and ovary at indicated floral development stages. (**A**) I_2_/KI staining of the anther sections of *NtMYC2a*-OE/RI and control plants at S4–S9; (**B**) Analyses of starch content in anther wall of *NtMYC2a*-OE/RI and control plants at indicated floral development stages. Data are shown as means ± SD, n = 10. * *p* < 0.05, ** *p* < 0.01 (Student’s *t*-test); (**C**) I_2_/KI staining of the ovaries of *NtMYC2a*-OE/RI and control plants at S9. Bar = 1 mm; (**D**) Analyses of starch content in ovary of *NtMYC2a*-OE/RI and control plants at indicated floral development stages. Results are shown as means ± SD, n = 3. Significant differences from the data of control plants are indicated: ** *p* < 0.01 (Student’s *t*-test).

The anther is composed of the anther wall and pollen, which, together with the ovary, determine the fertility of plant. We performed the histological assay to examine starch granules in the anther. The anther sections stained by I_2_/KI showed that the pollen grains of the *NtMYC2a*-silenced anther showed no or less staining at the S6 stage, while the pollen grains in the anthers of the *NtMYC2a*-OE and control plants were stained black at the same stage (Figure 3A). Similarly, the starch granules in the anther wall of the *NtMYC2a*-RI plants were significantly lower than that of the control and *NtMYC2a*-overexpressed plants at the S4 stage (Figure 3A). Compared to the S4 stage, the number of starch granules in the anther wall reduced in the control and *NtMYC2a*-OE plants at the S6 and S9 stages but increased to a much higher level in *NtMYC2a*-RI plants at the S6 stage (Figure 3A). To further verify the above results, we quantified the starch content of the anther wall of the *NtMYC2a*-OE, *NtMYC2a*-RI and control plants from S4 to S9. As shown in Figure 3B, the starch content in the anther wall of *NtMYC2a*-OE plants at S4 was significantly higher than that of the control and *NtMYC2a*-RI plants. However, at S6, the starch content in the anther wall of *NtMYC2a*-RI plants increased significantly, which was higher than that of the control and *NtMYC2a*-OE plants (Figure 3B). These results indicate that silencing *NtMYC2a* could delay starch accumulation in the anther wall.

Furthermore, the ovaries of *NtMYC2a*-OE, *NtMYC2a*-RI and control plants at the S9 stage were transected and iodine stained. As shown in Figure 3C, the ovaries of *NtMYC2a*-OE plants were more deeply stained than those of the control and *NtMYC2a*-RI plants. Consistently, compared with the control plants, the overexpression of *NtMYC2a* increased the starch content of the ovary at S4–S9 by 38.18%, 57.81% and 39.65%, respectively, while the starch content of the ovary of *NtMYC2a*-RI plants was similar to that of the control group, suggesting that overexpression of *NtMYC2a* could enhance the carbohydrate accumulation in tobacco ovaries (Figure 3D). These findings provide further evidence that NtMYC2a is involved in the regulation of carbohydrate metabolism in the anther wall and ovary.

### 3.4. NtMYC2a Regulates the Expression of Genes Related to Starch Metabolism during Flower Development

**Figure 4:**
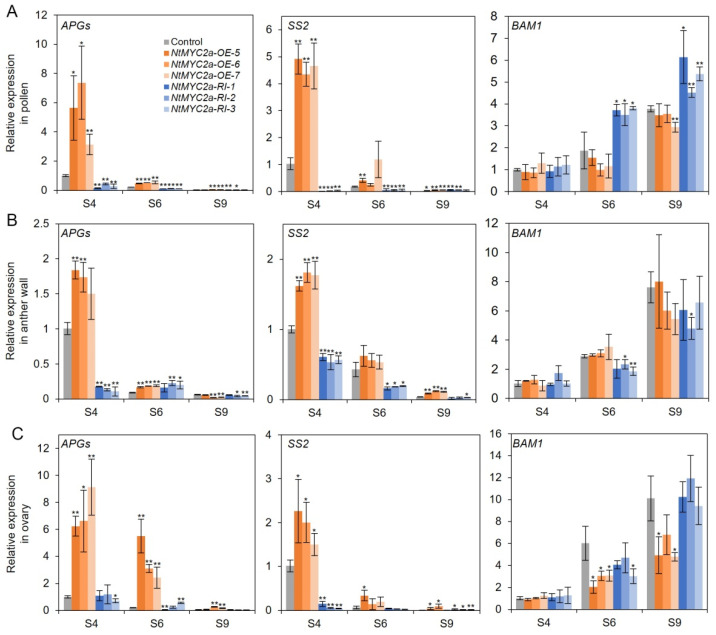
The transcriptions of *AGPs, SS2* and *BAM1* are regulated by *NtMYC2a* during flower development. Relative expression of *AGPs, SS2* and *BAM1* in pollen grains (**A**), anther walls (**B**) and ovaries (**C**) of *NtMYC2a-OE*, *NtMYC2a-RI* and control plants at S4, S6 and S9. For RT-qPCR assays, results are shown as means ± SD, n = 3. Significant differences from the data of control plant are indicated: * *p* < 0.05, ** *p* < 0.01 (Student’s *t*-test).

In order to determine the regulation of starch metabolic gene expression by NtMYC2a, we examined the expression of three starch metabolic genes, including *AGPs*, *SS2* and *BAM1*, in the pollen grains, anther walls and ovaries of *NtMYC2a*-OE, *NtMYC2a*-RI and control plants at S4–S9 [12,20,40]. As shown in Figure 4A, compared with control plants, the expression levels of *AGPs* and *SS2* in the pollen grains of *NtMYC2a*-RI plants were dramatically down-regulated at the S4 and S6 stages. Overexpression of *NtMYC2a* resulted in a sharp increase in the expression of *AGPs* and *SS2* at the same stages (Figure 4A). The expression of *BAM1* in the pollen grains of *NtMYC2a*-OE and *NtMYC2a*-RI plants was not significantly different from that of the control plants at S4 but was significantly up-regulated in the pollen grains of *NtMYC2a*-RI plants at S6 and S9 (Figure 4A). This suggests that NtMYC2a may play a positive role in starch synthesis and a negative role in starch degradation in pollen grains of tobacco.

Compared with the control plants, the expression of *AGPs* and *SS2* in the anther wall dramatically decreased in *NtMYC2a*-RI plants and significantly increased in *NtMYC2a*-OE plants at the S4 stage, which was consistent with the results of the I_2_/KI staining and starch quantification (Figure 3A,B and Figure 4B), indicating a positive role of NtMYC2a in starch synthesis in the anther wall at the early stages of flower development, while the relative expression level of *BAM1* did not show obvious changes in the anther walls of *NtMYC2a*-OE or *NtMYC2a*-RI plants at the S9 stage (Figure 4B). In the ovary, overexpression of *NtMYC2a* led to increased expression of *AGPs* and *SS2* at S4 and S6 and decreased expression of *BAM1* at S6 and S9 (Figure 4). This further confirmed the role of NtMYC2a in promoting starch synthesis and inhibiting starch degradation in the ovary. These results reveal that NtMYC2a affects the starch accumulation in the pollen grain, anther wall and ovary by regulating the expression of *AGPs*, *SS2* and *BAM1* during flower development.

### 3.5. Starch-Sugar Interconversion in Pollen, Anther Wall and Ovary of Tobacco Is Affected by NtMYC2a

The conversion of sugar to starch occurs throughout the growth process of plants and is essential for all plant growth [40]. Sugar is converted to starch at the early stages of flower development, and starch is degraded by AMYs and BAMs when the flower organs are mature, resulting in an increase of total soluble sugar in the plant [20].

**Figure 5:**
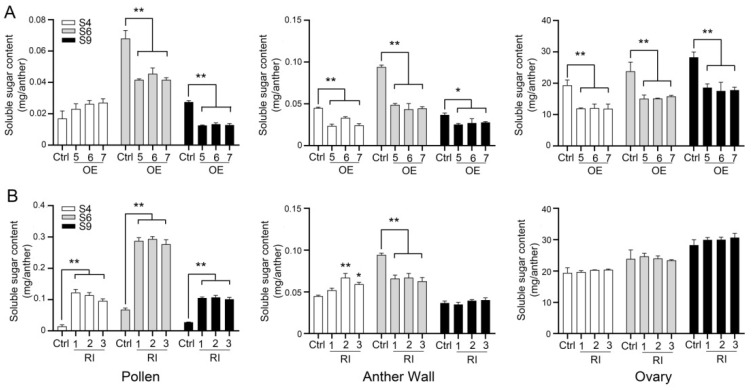
Analyses of the content of total soluble sugar at the indicated stages of floral development. The content of total soluble sugar in the pollen grains, anther walls and ovaries of *NtMYC2a*-OE (**A**), *NtMYC2a*-RI (**B**) and control plants were examined. Results are shown as means ± SD, n = 10, significant differences from the data of control plants are indicated: * *p* < 0.05, ** *p* < 0.01 (Student’s *t*-test).

To explore the role of NtMYC2a in the regulation of sugar-starch conversion, we quantified the total soluble sugars of the pollen, anther wall and ovary of *NtMYC2a*-OE, *NtMYC2a*-RI and control plants at S4–S9, respectively. As shown in Figure 5A, overexpression of *NtMYC2a* significantly decreased the level of total soluble sugars in pollen grains at S6 and S9 and in the anther walls and ovaries at S4–S9. The silencing of *NtMYC2a* led to a significant increase in the content of total soluble sugars in pollen grains at S4–S9 and in anther walls at S4, while it had no effect on the content of total soluble sugars in the ovaries (Figure 5B). These results indicate that, at S4, the main stage of starch synthesis, NtMYC2a promotes the conversion of sugar to starch in pollen, while, at S9, the hydrolysis of starch in pollen was inhibited by NtMYC2a, which is consistent with the results of the starch content quantification and RT-qPCR analysis (Figure 2C, Figure 4 and Figure 5). The quantification results of the starch content and total soluble sugar content in the anther wall and ovary also reveal that NtMYC2a plays an important regulatory role in promoting sugar to starch conversion at the S4 stage and inhibiting starch hydrolysis at the S9 stage (Figure 3B,D and Figure 5).

Taken together, these results suggest that NtMYC2a is involved in the regulation of the starch-sugar interconversion at different developmental stages of the pollen, anther wall and ovary.

### 3.6. Manipulation of NtMYC2a Expression Altered the Process of Pollen Maturation in Tobacco

**Figure 6:**
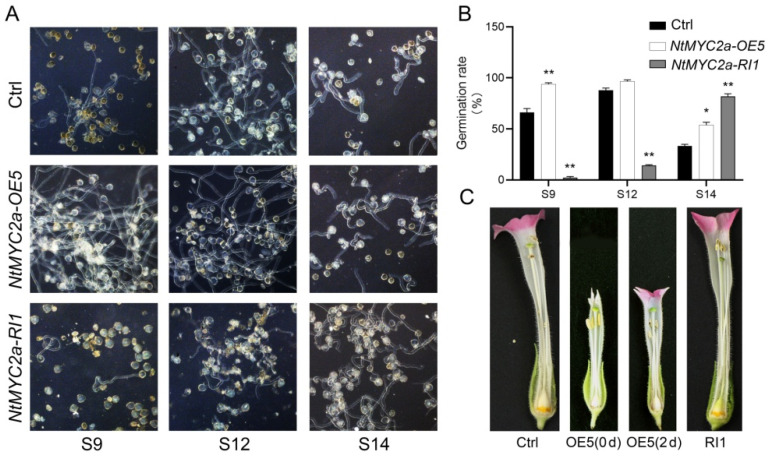
The pollen germination was delayed in the *NtMYC2a* knockdown plants. (**A**) Pollen germination of *NtMYC2a*-OE, *NtMYC2a*-RI and control plants at S9, S12 and S14; (**B**) Statistics of germination rate of *NtMYC2a*-OE, *NtMYC2a*-RI and control plants at indicated flower development stages. Results are shown as means ± SD, n = 100. Significant differences from the data of control plants are indicated: * *p* < 0.05, ** *p* < 0.01 (Student’s *t*-test); (**C**) The phenotypes of floral organs at S12. Ctrl, flower of control plants. OE5, flowers of *NtMYC2a*-OE plants at 0 days (0 d) and 2 days (2 d) after shedding. RI1, flower of *NtMYC2a*-RI plants.

We further investigated whether NtMYC2a regulates pollen germination or maturation. The pollen grains of *NtMYC2a*-OE/RI and control plants at S9, S12 and S14 were isolated from the anthers and cultivated on the germination medium for 10 h at 23 °C in the dark, and then the pollen germination rate was counted at S9, S12 and S14 stages. As shown in Figure 6A,B, in contrast to S9, the pollen germination rate of control and *NtMYC2a*-OE plants reached the peak at S12, which were 87.85% and 96.63% respectively. In general, the pollen germination rate of *NtMYC2a*-OE plants was higher than that of control plants at S9–S14, and the pollen germination rate of *NtMYC2a*-OE plants at S9 was close to or even higher than that of the control plants at S12, which indicates that the overexpression of *NtMYC2a* may lead to the premature maturation of tobacco pollen grains (Figure 6A,B). The pollen germination rates of *NtMYC2a*-RI plants at S9 and S12 were significantly lower than that of the control and *NtMYC2a*-OE plants, but it gradually increased and reached 81.77% at S14, which suggests that the silencing of *NtMYC2a* delayed the process of pollen maturation in tobacco (Figure 6A,B).

Additionally, the flower phenotypes of the control and transgenic plants at S12 were observed in this study. Compared with the control, the silencing of *NtMYC2a* had no impact on the growth and development of tobacco flowers, while overexpression of *NtMYC2a* led to smaller flowers and early abscission of floral organs (Figure 1D and Figure 6C). Further observation showed that the flowers of *NtMYC2a*-OE plants could continue to mature after abscission, and the pollen could germinate normally (Figure 6). These above results indicate that excessive or interference expression of *NtMYC2a* altered the process of pollen maturation, while given no impact on male fertility in tobacco.

## 4. Discussion

Carbohydrate metabolism provides the most critical nutrients for plant growth and development [41]. Starch, an insoluble, non-structural carbohydrate composed of α-glucose polymers, is one of the most important organic compounds in carbohydrate metabolism [41,42,43]. Recent study proved that starch metabolism plays an important role in shoot growth of bamboo [44]. In Arabidopsis, starch metabolism and accumulation are also associated with stamen and ovary development, suggesting a role of starch in plant reproductive development [45].

MYC2 functions as a core transcription factor in the JA signaling pathway. Various studies had reported the role of MYC2 in the secondary metabolism, stress tolerance, growth and development of plants [30]. MYC2 forms a complex with MYB21 to regulate linalool biosynthesis in flowers of *Freesia hybrida* and *Arabidopsis thaliana* [46]. AtMYC2 has also been demonstrated to regulate proline biosynthesis to assist Arabidopsis resist salt stress [47]. In addition, the biosynthesis of indole glucosinolate is negatively regulated by MYC2 through the JA signaling pathway in Arabidopsis. What is more, MYC2 may positively regulate JA-mediated insect resistance and oxidative stress tolerance by promoting the ascorbic acid redox cycle and flavonoid biosynthesis in Arabidopsis [48]. However, the role of MYC2 in regulating carbohydrate metabolism is rarely reported. Thus, in this study, we generated *NtMYC2a*-overexpressing (*NtMYC2a*-OE) and RNA-interference (NtMYC2a-RI) transgenic plants in tobacco (Figure 1). At S4–S9 of flower development, overexpression of *NtMYC2a* significantly increased the starch content, while the silencing of *NtMYC2a* resulted in a significant decrease of starch content in pollen grains, suggesting a positive role of NtMYC2a in starch accumulation in tobacco pollen (Figure 2). However, the regulation pattern of NtMYC2a on starch in the anther wall and ovary was a little different from that in pollen grains. Although overexpression of *NtMYC2a* increased starch content in the anther wall, similar to in pollen grains, at S4 and S6, starch content in the anther wall of *NtMYC2a*-RI plants at S6 and S9 did not show a significant decrease, especially at S6 (Figure 2C and Figure 3B). This implies a similar role of NtMYC2a as NtCOI1 in regulating starch accumulation in the anther wall of tobacco, which is consistent with the close interaction relationship between NtMYC2a and NtCOI1 in the JA signaling pathway [39]. Similarly, the overexpression of *NtMYC2a* increased the starch content of the ovary by 38%, 57.98% and 39.29% at S4, S6 and S9, respectively, while the interference of *NtMYC2a* did not lead to the decrease of the starch content of the ovary (Figure 3C,D). This suggests that NtMYC2a plays a sufficient but unnecessary role in starch accumulation in the anther wall and ovary. Interestingly, the accumulation of soluble sugars in the anther and ovary was also affected by NtMYC2a (Figure 5). Given that the changes in total soluble sugar contents in the pollen grain, anther wall, and ovary are accompanied by the accumulation and depletion of starch, we speculate that NtMYC2a may play a role in starch-sugar conversation, while the precise regulation mechanism of these processes needs to be further explored.

To explore the regulation mechanism of NtMYC2a on starch accumulation at the molecular level, we performed transcriptional analyses of *AGPs*, *SS2* and *BAM1*, which function in the starch metabolism of the *NtMYC2a*-OE/RI and control plants at the indicated stages (Figure 4) [39]. Results suggest that the active role of NtMYC2a in starch accumulation is probably accomplished through promoting the transcriptions of starch-synthesis-related genes (such as *AGPs* and *SS2*) at the early stages of floral development and inhibiting the expression of starch-degradation-related genes (*BAM1*) at the late stages of floral development in different flower organs. Whether NtMYC2a, as a bHLH transcription factor, binds to the promoters of these starch-metabolism-related genes to directly affect starch accumulation in the pollen, anther wall and ovary still needs to be verified.

Carbohydrates provide essential nutrients for plant reproduction and growth, and abnormal carbohydrate metabolism could result in pollen abortion [6,49]. Previous study revealed that different kinds and concentrations of sugar have different effects on pollen germination [22]. Glucose, fructose and mannose inhibit pollen germination in Arabidopsis [22]. In this study, we demonstrated that the accumulation of starch and sugar are regulated by NtMYC2a; thus, we speculate that NtMYC2a may affect pollen germination. As shown in Figure 6, compared with control plants, the germination rate of pollen grains was significantly increased in *NtMYC2a*-OE plants and significantly inhibited in *NtMYC2a*-RI plants at the early stage of flower development. Overexpression of *NtMYC2a* even accelerated the process of pollen germination and maturation (Figure 6A,B), while, at S14, the pollen germination rate of *NtMYC2a*-RI plants reached the normal level, which was even higher than that of the control and *NtMYC2a*-OE plants (Figure 6B). This suggests that NtMYC2a plays an active role in the on-schedule maturation of pollen grains, which is in accordance with the role of NtMYC2a on starch accumulation in pollen grains (Figure 2).

## 5. Conclusions

Carbohydrate supply is crucial in pollen development, and plant hormone JA plays an important regulator role in the process. MYC2, as a core regulator of the JA signaling pathway, also functions in pollen development, while the underlying mechanism is not clear. Our study revealed that tobacco transcription factor NtMYC2a participates in starch metabolism and starch-sugar conversion through regulating the expression of starch-metabolism-related genes, *AGPs*, *SS2* and *BAM1*, in the pollen, anther wall and ovary of plants. Meanwhile, NtMYC2a regulates the process of pollen maturation in tobacco. This suggests that NtMYC2a plays an important role in regulating carbohydrate metabolism in tobacco floral organs, thus affecting flower development, which provides a basis for further study on plant reproductive development.

## Data Availability

All the datasets included in this study have been presented within the manuscript and/or as Supplementary Files.

## Supplementary Materials

The Supplementary Materials online at https://www.mdpi.com/article/10.3390/plants11010017/s1, Supplementary Table S1: List of primers used in this study; Supplementary Figure S1: pBin-*NtMYC2a*-HA vector; Supplementary Figure S2: pBin-*NtMYC2aΔ*-RI vector.
