# Supplementary material for: bHLH Transcription Factor NtMYC2a Regulates Carbohydrate Metabolism during the Pollen Development of Tobacco (Nicotiana tabacum L. cv. TN90)
Source: Plants (Basel). 2021 Dec 22;11(1):17. doi: 10.3390/plants11010017 (PMC8747387; doi:10.3390/plants11010017)
Supplement: Supplementary file 1 [file plants-11-00017-s001.zip › plants-1495281-supplementary.pdf]

Supplementary Material

bHLH Transcription Factor NtMYC2a Regulates the Carbohydrate Metabolism During Pollen Development in Tobacco (*Nicotiana tabacum* L. cv. TN90)

Shiquan Bian [1], Tian Tian [1], Yongqiang Ding [1], Ning Yan [1], Chunkai Wang [1], Ning Fang [1], Yanhua Liu [1], Zhongfeng Zhang [1] and Hongbo Zhang [1,*]

[1] Tobacco Research Institute of Chinese Academy of Agricultural Sciences, Qingdao 266101, China; bsq20200130@163.com (B.S.); tiantian01@caas.cn (T.T.); dyq910420@163.com (D.Y); yanning@caas.cn (N.Y.); wangchunkai1990@outlook.com (W.C.); fangning010@163.com (F.N); liuyanhua@caas.cn (L.Y); zhangzhongfeng@caas.cn (Z.Z.)

* Correspondence: zhanghongbo@caas.cn (Z.H.)

Supplementary Table S1: List of primers used in this study

Gene	GenBank Accession	Primers	Primer Use
NtMYC2a	LOC107820916	5′-aaaCCGCGGATGACTGATTACAGCTTACCCA-3′<br>5′-GCGTGTTTCAGCAACTCTGGAT-3′	For constructing pBin- *NtMYC2a*-HA vector
NtMYC2a	LOC107820916	5′-aaaCCGCGGATGACTGATTACAGCTTACCCA-3′<br>5′-GGAAGCCCACTTCCGTTAACAAAC-3′	For constructing pBin- *NtMYC2aΔ*-RI vector
NtMYC2a	LOC107820916	5′-TCACCCATTTCTCTCTCTCTCTC-3′<br>5′-GAGGTAACAGCAGCAGTAGTAG-3′	For RT-qPCR
AGPs	AB018190	5′-GCTTTCTACAATGCCAATTTGG-3′<br>5′-ACTGTCTGTGACATCGGCAT-3′	
SS2	JX264160	5′-GGCATACTGCTTTATTGCCA-3′<br>5′-TGGTGGAAGATCTACGTATGAAA-3′	
BAM1	KF420482	5′-AGCGTGTCAAGCACTAATGG-3′<br>5′-CTGTCCAACGGCATCATAAC-3′	
NtActin	X63603	5′-CCACACAGGTGTGATGGTTG-3′<br>5′-GTGGCTAACACCATCACCAG-3′	

Supplementary Figure S1: pBin-*NtMYC2a*-HA vector

Vector map	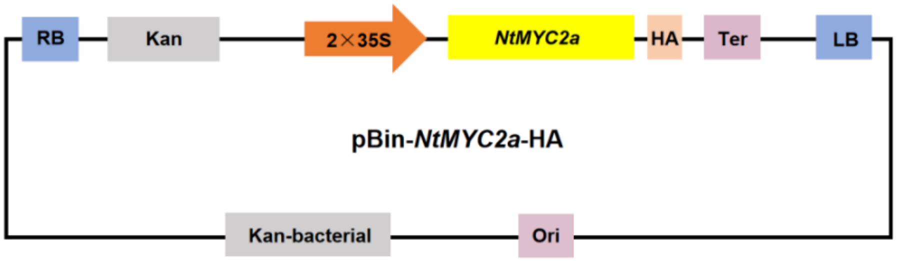
Vector sequence	TGAGCGTCGCAAAGGCGCTCGGTCTTGCCTTGCTCGTCGGTGATGTACTTCACCAGCTCCGCGAAGTCGCTCTTCTTGATGGAGCGCATGGGGACGTGCTTGGCAATCACGCGCA<br>CCCCCCGGCCGTTTTAGCGGCTAAAAAAGTCATGGCTCTGCCCTCGGGCGGACCACGCCCATCATGACCTTGCCAAGCTCGTCCTGCTTCTCTTCGATCTTCGCCAGCAGGGCGA<br>GGATCGTGGCATCACCGAACCGCGCCGTGCGCGGGTCGTCGGTGAGCCAGAGTTTCAGCAGGCCGCCCAGGCGGCCCAGGTCGCCATTGATGCGGGCCAGCTCGCGGACGTGCT<br>CATAGTCCACGACGCCCGTGATTTTGTAGCCCTGGCCGACGGCCAGCAGGTAGGCCGACAGGCTCATGCCGGCCGCCGCCGCCTTTTCCTCAATCGCTCTTCGTTCGTCTGGAAG<br>GCAGTACACCTTGATAGGTGGGCTGCCCTTCCTGGTTGGCTTGGTTTCATCAGCCATCCGCTTGCCCTCATCTGTTACGCCGGCGGTAGCCGGCCAGCCTCGCAGAGCAGGATTCC<br>CGTTGAGCACCGCCAGGTGCGAATAAGGGACAGTGAAGAAGGAACACCCGCTCGCGGGTGGGCCTACTTCACCTATCCTGCCCGGCTGACGCCGTTGGATACACCAAGGAAAGT<br>CTACACGAACCCTTTGGCAAAATCCTGTATATCGTGCGAAAAAGGATGGATATACCGAAAAAATCGCTATAATGACCCCGAAGCAGGGTTATGCAGCGGAAAAGCGCCACGCTTCC<br>CGAAGGGAGAAAGGCGGACAGGTATCCGGTAAGCGGCAGGGTCGGAACAGGAGAGCGCACGAGGGAGCTTCCAGGGGGAAACGCCTGGTATCTTTATAGTCCTGTCGGGTTTC<br>GCCACCTCTGACTTGAGCGTCGATTTTTGTGATGCTCGTCAGGGGGGCGGAGCCTATGGAAAAACGCCAGCAACGCGGCCTTTTTACGGTTCCTGGCCTTTTGCTGGCCTTTTGCT<br>CACATGTTCTTTCCTGCGTTATCCCCTGATTCTGTGGATAACCGTATTACCGCCTTTGAGTGAGCTGATACCGCTCGCCGCAGCCGAACGACCGAGCGCAGCGAGTCAGTGAGCGA<br>GGAAGCGGAAGAGCGCCAGAAGGCCGCCAGAGAGGCCGAGCGCGGCCGTGAGGCTTGGACGCTAGGGCAGGGCATGAAAAAGCCCGTAGCGGGCTGCTACGGGCGTCTGACG<br>CGGTGGAAAGGGGGAGGGGATGTTGTCTACATGGCTCTGCTGTAGTGAGTGGGTTGCGCTCCGGCAGCGGTCCTGATCAATCGTCACCCTTTCTCGGTCCTTCAACGTTCCTGAC<br>AACGAGCCTCCTTTTCGCCAATCCATCGACAATCACCGCGAGTCCCTGCTCGAACGCTGCGTCCGGACCGGCTTCGTCGAAGGCGTCTATCGCGGCCCGCAACAGCGGCGAGAG<br>CGGAGCCTGTTCAACGGTGCCGCCGCGCTCGCCGGCATCGCTGTCGCCGGCCTGCTCCTCAAGCACGGCCCCAACAGTGAAGTAGCTGATTGTCATCAGCGCATTGACGGCGTCC<br>CCGGCCGAAAAACCCGCCTCGCAGAGGAAGCGAAGCTGCGCGTCGGCCGTTTCCATCTGCGGTGCGCCCGGTCGCGTGCCGGCATGGATGCGCGCGCCATCGCGGTAGGCGAGC<br>AGCGCCTGCCTGAAGCTGCGGGCATTCCCGATCAGAAATGAGCGCCAGTCGTCGTCGGCTCTCGGCACCGAATGCGTATGATTCTCCGCCAGCATGGCTTCGGCCAGTGCGTCGA<br>GCAGCGCCCGCTTGTTCCTGAAGTGCCAGTAAAGCGCCGGCTGCTGAACCCCCAACCGTTCCGCCAGTTTGCGTGTCGTCAGACCGTCTACGCCGACCTCGTTCAACAGGTCCA<br>GGGCGGCACGGATCACTGTATTCGGCTGCAACTTTGTCATGCTTGACACTTTATCACTGATAAACATAATATGTCCACCAACTTATCAGTGATAAAGAATCCGCGCGTTCAATCGGA<br>CCAGCGGAGGCTGGTCCGGAGGCCAGACGTGAAACCCAACATACCCCTGATCGTAATTCTGAGCACTGTCGCGCTCGACGCTGTCGGCATCGGCCTGATTATGCCGGTGCTGCCG<br>GGCCTCCTGCGCGATCTGGTTCACTCGAACGACGTCACCGCCCACTATGGCATTCTGCTGGCGCTGTATGCGTTGGTGCAATTTGCCTGCGCACCTGTGCTGGGCGCGCTGTCGGA<br>TCGTTTCGGGCGGCGGCCAATCTTGCTCGTCTCGCTGGCCGGCGCCAGATCTGGGGAACCCTGTGGTTGGCATGCACATACAAATGGACGAACGGATAAACCTTTTCACGCCCTTT<br>TAAATATCCGATTATTCTAATAAACGCTCTTTTCTCTTAGGTTTACCCGCCAATATATCCTGTCAAACACTGATAGTTTAAACTGAAGGCGGGAAACGACAATCTGATCATGAGCGGA<br>GAATTAAGGGAGTCACGTTATGACCCCCGCCGATGACGCGGGACAAGCCGTTTTACGTTTGGAACTGACAGAACCGCAACGTTGAAGGAGCCACTCAGCCGCGGGTTTCTGGAG<br>TTTAATGAGCTAAGCACATACGTCAGAAACCATTATTGCGCGTTCAAAAGTCGCCTAAGGTCACTATCAGCTAGCAAATATTTCTTGTCAAAAATGCTCCACTGACGTTCCATAAAT<br>TCCCCTCGGTATCCAATTAGAGTCTCATATTCACTCTCAATCCAAATAATCTGCACCGGATCTGGATCGTTTCGCATGATTGAACAAGATGGATTGCACGCAGGTTCTCCGGCCGCTT<br>GGGTGGAGAGGCTATTCGGCTATGACTGGGCACAACAGACAATCGGCTGCTCTGATGCCGCCGTGTTCCGGCTGTCAGCGCAGGGGCGCCCGGTTCTTTTTGTCAAGACCGACCT<br>GTCCGGTGCCCTGAATGAACTGCAGGACGAGGCAGCGCGGCTATCGTGGCTGGCCACGACGGGCGTTCCTTGCGCAGCTGTGCTCGACGTTGTCACTGAAGCGGGAAGGGACTG<br>GCTGCTATTGGGCGAAGTGCCGGGGCAGGATCTCCTGTCATCTCACCTTGCTCCTGCCGAGAAAGTATCCATCATGGCTGATGCAATGCGGCGGCTGCATACGCTTGATCCGGCTA<br>CCTGCCCATTCGACCACCAAGCGAAACATCGCATCGAGCGAGCACGTACTCGGATGGAAGCCGGTCTTGTCGATCAGGATGATCTGGACGAAGAGCATCAGGGGCTCGCGCCAG<br>CCGAACTGTTCGCCAGGCTCAAGGCGCGCATGCCCGACGGCGATGATCTCGTCGTGACCCATGGCGATGCCTGCTTGCCGAATATCATGGTGGAAAATGGCCGCTTTTCTGGATTC<br>ATCGACTGTGGCCGGCTGGGTGTGGCGGACCGCTATCAGGACATAGCGTTGGCTACCCGTGATATTGCTGAAGAGCTTGGCGGCGAATGGGCTGACCGCTTCCTCGTGCTTTACGG<br>TATCGCCGCTCCCGATTCGCAGCGCATCGCCTTCTATCGCCTTCTTGACGAGTTCTTCTGAGCGGGACTCTGGGGTTCGAAATGACCGACCAAGCGACGCCCAACCTGCCATCACG<br>AGATTTCGATTCCACCGCCGCCTTCTATGAAAGGTTGGGCTTCGGAATCGTTTTCCGGGACGCCGGCTGGATGATCCTCCAGCGCGGGGATCTCATGCTGGAGTTCTTCGCCCACG<br>GGATCTCTGCGGAACAGGCGGTCGAAGGTGCCGATATCATTACGACAGCAACGGCCGACAAGCACAACGCCACGATCCTGAGCGACAATATGATCGGGCCCGGCGTCCACATCA<br>ACGGCGTCGGCGGCGACTGCCCAGGCAAGACCGAGATGCACCGCGATATCTTGCTGCGTTCGGATATTTTCGTGGAGTTCCCGCCACAGACCCGGATGATCCCCGATCGTTCAAA<br>CATTTGGCAATAAAGTTTCTTAAGATTGAATCCTGTTGCCGGTCTTGCGATGATTATCATATAATTTCTGTTGAATTACGTTAAGCATGTAATAATTAACATGTAATGCATGACGTTATT<br>TATGAGATGGGTTTTTATGATTAGAGTCCCGCAATTATACATTTAATACGCGATAGAAAACAAAATATAGCGCGCAAACTAGGATAAATTATCGCGCGCGGTGTCATCTATGTTACTAG<br>ATCGGGCCTCCTGTCAATGCTGGCGGCGGCTCTGGTGGTGGTTCTGGTGGCGGCTCTGAGGGTGGTGGCTCTGAGGGTGGCGGTTCTGAGGGTGGCGGCTCTGAGGGAGGCGGT<br>TCCGGTGGTGGCTCTGGTTCCGGTGATTTTGATTATGAAAAGATGGCAAACGCTAATAAGGGGGCTATGACCGAAAATGCCGATGAAAACGCGCTACAGTCTGACGCTAAAGGCA<br>AACTTGATTCTGTCGCTACTGATTACGGTGCTGCTATCGATGGTTTCATTGGTGACGTTTCCGGCCTTGCTAATGGTAATGGTGCTACTGGTGATTTTGCTGGCTCTAATTCCCAAAT<br>GGCTCAAGTCGGTGACGGTGATAATTCACCTTTAATGAATAATTTCCGTCAATATTTACCTTCCCTCCCTCAATCGGTTGAATGTCGCCCTTTTGTCTTTGGCCCAATACGCAAACCG<br>CCTCTCCCCGCGCGTTGGCCGATTCATTAATGCAGCTGGCACGACAGGTTTCCCGACTGGAAAGCGGGCAGTGAGCGCAACGCAATTAATGTGAGTTAGCTCACTCATTAGGCAC<br>CCCAGGCTTTACACTTTATGCTTCCGGCTCGTATGTTGTGTGGAATTGTGAGCGGATAACAATTTCACACAGGAAACAGCTATGACCATGATTACGCCAAGCTTGATATCGAATTCC<br>TGCAGGTCAACATGGTGGAGCACGACACACTTGTCTACTCCAAAAATATCAAAGATACAGTCTCAGAAGACCAAAGGGCAATTGAGACTTTTCAACAAAGGGTAATATCCGGAAA<br>CCTCCTCGGATTCCATTGCCCAGCTATCTGTCACTTTATTGTGAAGATAGTGGAAAAGGAAGGTGGCTCCTACAAATGCCATCATTGCGATAAAGGAAAGGCCATCGTTGAAGATG<br>CCTCTGCCGACAGTGGTCCCAAAGATGGACCCCCACCCACGAGGAGCATCGTGGAAAAAGAAGACGTTCCAACCACGTCTTCAAAGCAAGTGGATTGATGTGATAACATGGTGG<br>AGCACGACACACTTGTCTACTCCAAAAATATCAAAGATACAGTCTCAGAAGACCAAAGGGCAATTGAGACTTTTCAACAAAGGGTAATATCCGGAAACCTCCTCGGATTCCATTG<br>CCCAGCTATCTGTCACTTTATTGTGAAGATAGTGGAAAAGGAAGGTGGCTCCTACAAATGCCATCATTGCGATAAAGGAAAGGCCATCGTTGAAGATGCCTCTGCCGACAGTGGTC<br>CCAAAGATGGACCCCCACCCACGAGGAGCATCGTGGAAAAAGAAGACGTTCCAACCACGTCTTCAAAGCAAGTGGATTGATGTGATATCTCCACTGACGTAAGGGATGACGCAC<br>AATCCCACTATCCTTCGCAAGACCCTTCCTCTATATAAGGAAGTTCATTTCATTTGGAGAGGACCTCGAGAATTCTCAACACAACATATACAAAACAAACGAATCTCAAGCAATCAA<br>GCATTCTACTTCTATTGCAGCAATTTAAATCATTTCTTTTAAAGCAAAAGCAATTTTCTGAAAATTTTCACCATTTACGAACGATAGGCCTAGGACAAGTTTGTACAAAAAAGCAGG<br>CTCCGCGGATGACTGATTACAGCTTACCCACCATGAATTTGTGGAATACTAGTGGTACTACCGATGACAACGTTTCTATGATGGAATCTTTTATGTCTTCTGATCTCACTTCATTTGG<br>GCTACTTCTAATTCTACTACTGCTGCTGTTACCTCTAATTCTAATCTTATTCCAGTTAATACCCTAACTGTTCTTCTTCCGTCTTCTTGTGCTTCTACTGTCACAGCTGTGGCTGTCGAT<br>GCTTCAAAATCCATGTCTTTTTTCAACCAAGAAACTCTTCAGCAGCGTCTTCAAACCCTCATTGATGGTGCTCGTGAGACGTGGACCTATGCCATCTTTTGGCAGTCATCCGTCGTT<br>GATTTATCGAGTCCGTTTGTGTTGGGCTGGGGAGATGGTTACTACAAAGGTGAAGAAGATAAAGCCAATAGGAAATTAGCTGTTTCTTCTCCTGCTTATATTGCTGAGCAAGAACA<br>CCGAAAAAAGGTTCTCCGGGAGCTGAATTCGTTGATCTCCGGCACGCAAACCGGCACTGATGATGCCGTCGATGAAGAAGTTACCGACACTGAATGGTTCTTCCTTATTTCCATGA<br>CCCAATCGTTTGTTAACGGAAGTGGGCTTCCGGGTCAGGCCTTATACAATTCCAGCCCTATTTGGGTCGCCGGAGCAGAGAAATTGGCAGCTTCCCACTGCGAACGGGCTCGGCA<br>GGCCCAGGGATTCGGGCTTCAGACGATGGTTTGTATTCCTTCAGCAAACGGCGTGGTTGAATTGGGCTCCACGGAGTTGATAATCCAGAGTTGTGATCTCATGAACAAGGTTAGAG<br>TATTGTTTAACTTCAATAATGATTTGGGCTCTGGTTCGTGGGCTGTGCAGCCCGAGAGCGATCCGTCCGCTCTTTGGCTCACTGATCCATCGTCTGCAGCTGTAGAAGTCCAAGATT<br>TAAATACAGTTAAGGCAAATTCAGTTCCATCAAGTAATAGTAGTAAGCAAGTTGTGTTTGATAATGAGAATAATGGTCACAGTTCTGATAATCAGCAACAGCAGCATTCTAAGCATG<br>AAACACAAGGATTTTTCACAAGGGAGTTGAATTTTTCAGAATTTGGGTTTGATGGAAGTAGTAATAATAGGAATGGGAATTCATCACTTTCTTGCAAGCCAGAGTCGGGGGAAATC<br>TTGAATTTTGGTGATAGTACTAAGAAAAGTGCAAATGGGAACTTATTTTCGGGTCAGTCCCATTTTGGGGCAGGGGAGGAGAATAAGAACAAGAAAAGGTCACCTGCTTCCAGAG<br>GAAGCAATGAAGAAGGAATGCTTTCATTTGTTTCGGGTACAATCTTGCCTGCAGCTTCTGGTGCGATGAAGTCAAGTGGAGGTGTAGGTGAAGACTCTGATCATTCGGATCTTGAG<br>GCCTCAGTGGTGAAAGAAGCTGAAAGTAGTAGAGTTGTAGAACCCGAAAAGAGGCCAAAGAAGCGAGGAAGGAAGCCAGCAAATGGACGGGAGGAACCTTTGAATCACGTCG<br>AAGCAGAGAGGCAAAGGAGAGAGAAATTAAACCAAAGGTTCTACGCATTAAGAGCTGTTGTTCCGAATGTGTCCAAGATGGACAAGGCATCACTGCTTGGAGATGCAATTTCATA<br>TATTAATGAGCTGAAGTTGAAGCTTCAAAATACAGAAACAGATAGAGAAGAATTGAAGAGCCAAATAGAAGATTTAAAGAAAGAATTAGTTAGTAAAGACTCAAGGCGCCCTGGT<br>CCTCCACCATCAAATCATGATCACAAGATGTCTAGCCATACTGGAAGCAAGATTGTAGACGTGGATATAGATGTTAAGATAATTGGATGGGATGCGATGATTCGTATACAATGTAATA<br>AAAAGAATCATCCAGCTGCAAGGTTAATGGTAGCCCTCAAGGAGTTAGATCTAGATGTGCACCATGCCAGTGTTTCAGTGGTGAACGATTTGATGATCCAACAAGCCACTGTGAA<br>AATGGGTAGCAGACTTTACACGGAAGAGCAACTTAGGATAGCATTGACATCCAGAGTTGCTGAAACACGCCGCGCCGACCCAGCTTTCTTGTACAAAGTGGTTGATAACAGCGGG<br>TTAATTAACATCTTTTACCCATACGATGTTCCTGACTATGCGGGCTATCCCTATGACGTCCCGGACTATGCAGGATCCTATCCATATGACGTTCCAGATTACGCTGCTCAGTGCAGCGC<br>TTAGAGCCTAGTTCTAGAGTCCGCAAAAATCACCAGTCTCTCTCTACAAATCTATCTCTCTCTATTTTTCTCCAGAATAATGTGTGAGTAGTTCCCAGATAAGGGAATTAGGGTTCTT<br>ATAGGGTTTCGCTCATGTGTTGAGCATATAAGAAACCCTTAGTATGTATTTGTATTTGTAAAATACTTCTATCAATAAAATTTCTAATTCCTAAAACCAAAATCCAGTGACCTGCAGCC<br>CGGGGGATCCACTAGAGCGGCCGCCACCGGGTACCGAGCTCGAATTCACTGGCCGTCGTTTTACAACGTCGTGACTGGGAAAACCCTGGCGTTACCCAACTTAATCGCCTTGCAG<br>CACATCCCCCTTTCGCCAGCTGGCGTAATAGCGAAGAGGCCCGCACCGATCGCCCTTCCCAACAGTTGCGCAGCCTGAATGGCGCCCGCTCCTTTCGCTTTCTTCCCTTCCTTTCT<br>CGCCACGTTCGCCGGCTTTCCCCGTCAAGCTCTAAATCGGGGGCTCCCTTTAGGGTTCCGATTTAGTGCTTTACGGCACCTCGACCCCAAAAAACTTGATTTGGGTGATGGTTCAC<br>GTAGTGGGCCATCGCCCTGATAGACGGTTTTTCGCCCTTTGACGTTGGAGTCCACGTTCTTTAATAGTGGACTCTTGTTCCAAACTGGAACAACACTCAACCCTATCTCGGGCTATT<br>CTTTTGATTTATAAGGGATTTTGCCGATTTCGGAACCACCATCAAACAGGATTTTCGCCTGCTGGGGCAAACCAGCGTGGACCGCTTGCTGCAACTCTCTCAGGGCCAGGCGGTGA<br>AGGGCAATCAGCTGTTGCCCGTCTCACTGGTGAAAAGAAAAACCACCCCAGTACATTAAAAACGTCCGCAATGTGTTATTAAGTTGTCTAAGCGTCAATTTGTTTACACCACAATA<br>TATCCTGCCACCAGCCAGCCAACAGCTCCCCGACCGGCAGCTCGGCACAAAATCACCACTCGATACAGGCAGCCCATCAGTCCGGGACGGCGTCAGCGGGAGAGCCGTTGTAAG<br>GCGGCAGACTTTGCTCATGTTACCGATGCTATTCGGAAGAACGGCAACTAAGCTGCCGGGTTTGAAACACGGATGATCTCGCGGAGGGTAGCATGTTGATTGTAACGATGACAGA<br>GCGTTGCTGCCTGTGATCAAATATCATCTCCCTCGCAGAGATCCGAATTATCAGCCTTCTTATTCATTTCTCGCTTAACCGTGACAGGCTGTCGATCTTGAGAACTATGCCGACATAA<br>TAGGAAATCGCTGGATAAAGCCGCTGAGGAAGCTGAGTGGCGCTATTTCTTTAGAAGTGAACGTTGACGATATCAACTCCCCTATCCATTGCTCACCGAATGGTACAGGTCGGGGA<br>CCCGAAGTTCCGACTGTCGGCCTGATGCATCCCCGGCTGATCGACCCCAGATCTGGGGCTGAGAAAGCCCAGTAAGGAAACAACTGTAGGTTCGAGTCGCGAGATCCCCCGGAA<br>CCAAAGGAAGTAGGTTAAACCCGCTCCGATCAGGCCGAGCCACGCCAGGCCGAGAACATTGGTTCCTGTAGGCATCGGGATTGGCGGATCAAACACTAAAGCTACTGGAACGAG<br>CAGAAGTCCTCCGGCCGCCAGTTGCCAGGCGGTAAAGGTGAGCAGAGGCACGGGAGGTTGCCACTTGCGGGTCAGCACGGTTCCGAACGCCATGGAAACCGCCCCCGCCAGGC<br>CCGCTGCGACGCCGACAGGATCTAGCGCTGCGTTTGGTGTCAACACCAACAGCGCCACGCCCGCAGTTCCGCAAATAGCCCCCAGGACCGCCATCAATCGTATCGGGCTACCTAG<br>CAGAGCGGCAGAGATGAACACGACCATCAGCGGCTGCACAGCGCCTACCGTCGCCGCGACCCCGCCCGGCAGGCGGTAGACCGAAATAAACAACAAGCTCCAGAATAGCGAAA

	TATTAAGTGCGCCGAGGATGAAGATGCGCATCCACCAGATTCCCGTTGGAATCTGTCGGACGATCATCACGAGCAATAAACCCGCCGGCAACGCCCGCAGCAGCATACCGGCGAC<br>CCCTCGGCCTCGCTGTTCGGGCTCCACGAAAACGCCGGACAGATGCGCCTTGTGAGCGTCCTTGGGGCCGTCCTCCTGTTTGAAGACCGACAGCCCAATGATCTCGCCGTCGATG<br>TAGGCGCCGAATGCCACGGCATCTCGCAACCGTTCAGCGAACGCCTCCATGGGCTTTTTCTCCTCGTGCTCGTAAACGGACCCGAACATCTCTGGAGCTTTCTTCAGGGCCGACA<br>ATCGGATCTCGCGGAAATCCTGCACGTCGGCCGCTCCAAGCCGTCGAATCTGAGCCTTAATCACAATTGTCAATTTTAATCCTCTGTTTATCGGCAGTTCGTAGAGCGCGCCGTGCG<br>TCCCGAGCGATACTGAGCGAAGCAAGTGCGTCGAGCAGTGCCCGCTTGTTCCTGAAATGCCAGTAAAGCGCTGGCTGCTGAACCCCCAGCCGGAACTGACCCCACAAGGCCCTA<br>GCGTTTGCAATGCACCAGGTCATCATTGACCCAGGCGTGTTCCACCAGGCCGCTGCCTCGCAACTCTTCGCAGGCTTCGCCGACCTGCTCGCGCCACTTCTTCACGCGGTGGAA<br>TCCGATCCGCACATGAGGCGGAAGGTTTCCAGCTTGAGCGGGTACGGCTCCCGGTGCGAGCTGAAATAGTCGAACATCCGTCGGGCCGTCGGCGACAGCTTGCGGTACTTCTCCC<br>ATATGAATTTCGTGTAGTGGTCGCCAGCAAACAGCACGACGATTTCCTCGTCGATCAGGACCTGGCAACGGGACGTTTTCTTGCCACGGTCCAGGACGCGGAAGCGGTGCAGCAG<br>CGACACCGATTCCAGGTGCCCAACGCGGTCGGACGTGAAGCCCATCGCCGTCGCCTGTAGGCGCGACAGGCATTCCTCGGCCTTCGTGTAATACCGGCCATTGATCGACCAGCCC<br>AGGTCCTGGCAAAGCTCGTAGAACGTGAAGGTGATCGGCTCGCCGATAGGGGTGCGCTTCGCGTACTCCAACACCTGCTGCCACACCAGTTCGTCATCGTCGGCCCGCAGCTCGA<br>CGCCGGTGTAGGTGATCTTCACGTCCTTGTTGACGTGGAAAATGACCTTGTTTTGCAGCGCCTCGCGCGGGATTTTCTTGTTGCGCGTGGTGAACAGGGCAGAGCGGGCCGTGTC<br>GTTTGGCATCGCTCGCATCGTGTCCGGCCACGGCGCAATATCGAACAAGGAAAGCTGCATTTCCTTGATCTGCTGCTTCGTGTGTTTCAGCAACGCGGCCTGCTTGGCCTCGCTGA<br>CCTGTTTTGCCAGGTCCTCGCCGGCGGTTTTTCGCTTCTTGGTCGTCATAGTTCCTCGCGTGTCGATGGTCATCGACTTCGCCAAACCTGCCGCCTCCTGTTCGAGACGACGCGAA<br>CGCTCCACGGCGGCCGATGGCGCGGGCAGGGCAGGGGGAGCCAGTTGCACGCTGTCGCGCTCGATCTTGGCCGTAGCTTGCTGGACCATCGAGCCGACGGACTGGAAGGTTTCG<br>CGGGGCGCACGCATGACGGTGCGGCTTGCGATGGTTTCGGCATCCTCGGCGGAAAACCCCGCGTCGATCAGTTCTTGCCTGTATGCCTTCCGGTCAAACGTCCGATTCATTCACCC<br>TCCTTGCGGGATTGCCCCGACTCACGCCGGGGCAATGTGCCCTTATTCCTGATTTGACCCGCCTGGTGCCTTGGTGTCCAGATAATCCACCTTATCGGCAATGAAGTCGGTCCCGTA<br>GACCGTCTGGCCGTCCTTCTCGTACTTGGTATTCCGAATCTTGCCCTGCACGAATACCAGCGACCCCTTGCCCAAATACTTGCCGTGGGCCTCGGCCTGAGAGCCAAAACACTTGA<br>TGCGGAAGAAGTCGGTGCGCTCCTGCTTGTCGCCGGCATCGTTGCGCCACATCTAGGTACTAAAACAATTCATCCAGTAAAATATAATATTTTATTTTCTCCCAATCAGGCTTGATCC<br>CCAGTAAGTCAAAAAATAGCTCGACATACTGTTCTTCCCCGATATCCTCCCTGATCGACCGGACGCAGAAGGCAATGTCATACCACTTGTCCGCCCTGCCGCTTCTCCCAAGATCAA<br>TAAAGCCACTTACTTTGCCATCTTTCACAAAGATGTTGCTGTCTCCCAGGTCGCCGTGGGAAAAGACAAGTTCCTCTTCGGGCTTTTCCGTCTTTAAAAAATCATACAGCTCGCGC<br>GGATCTTTAAATGGAGTGTCTTCTTCCCAGTTTTCGCAATCCACATCGGCCAGATCGTTATTCAGTAAGTAATCCAATTCGGCTAAGCGGCTGTCTAAGCTATTCGTATAGGGACAAT<br>CCGATATGTCGATGGAGTGAAAGAGCCTGATGCACTCCGCATACAGCTCGATAATCTTTTCAGGGCTTTGTTCATCTTCATACTCTTCCGAGCAAAGGACGCCATCGGCCTCACTCA<br>TGAGCAGATTGCTCCAGCCATCATGCCGTTCAAAGTGCAGGACCTTTGGAACAGGCAGCTTTCCTTCCAGCCATAGCATCATGTCCTTTTCCCGTTCCACATCATAGGTGGTCCCTT<br>TATACCGGCTGTCCGTCATTTTTAAATATAGGTTTTCATTTTCTCCCACCAGCTTATATACCTTAGCAGGAGACATTCCTTCCGTATCTTTTACGCAGCGGTATTTTTCGATCAGTTTTT<br>TCAATTCCGGTGATATTCTCATTTTAGCCATTTATTATTTCCTTCCTCTTTTCTACAGTATTTAAAGATACCCCAAGAAGCTAATTATAACAAGACGAACTCCAATTCACTGTTCCTTGC<br>ATTCTAAAACCTTAAATACCAGAAAACAGCTTTTTCAAAGTTGTTTTCAAAGTTGGCGTATAACATAGTATCGACGGAGCCGATTTTGAAACCACAATTATGGGTGATGCTGCCAAC<br>TTACTGATTTAGTGTATGATGGTGTTTTTGAGGTGCTCCAGTGGCTTCTGTGTCTATCAGCTGTCCCTCCTGTTCAGCTACTGACGGGGTGGTGCGTAACGGCAAAAGCACCGCCG<br>GACATCAGCGCTATCTCTGCTCTCACTGCCGTAAAACATGGCAACTGCAGTTCACTTACACCGCTTCTCAACCCGGTACGCACCAGAAAATCATTGATATGGCCATGAATGGCGTT<br>GGATGCCGGGCAACAGCCCGCATTATGGGCGTTGGCCTCAACACGATTTTACGTCACTTAAAAAACTCAGGCCGCAGTCGGTAACCTCGCGCATACAGCCGGGCAGTGACGTCAT<br>CGTCTGCGCGGAAATGGACGAACAGTGGGGCTATGTCGGGGCTAAATCGCGCCAGCGCTGGCTGTTTTACGCGTTGGGAGCTCTCCCATATGGTCGACCTGCAGGCGGCCGCGAA<br>TTCACTAGTGATACCCCATATGTGATCAATTCGGGCACGAACCCAGTGGACATAAGCCTCGTTCGGTTCGTAAGCTGTAATGCAAGTAGCGTAACTGCCGTCACGCAACTGGTCCA<br>GAACCTTGACCGAACGCAGCGGTGGTAACGGCGCAGTGGCGGTTTTCATGGCTTCTTGTTATGACATGTTTTTTTGGGGTACAGTCTATGCCTCGGGCATCCAAGCAGCAAGCGCG<br>TTACGCCGTGGGTCGATGTTTGATGTTATGGAGCAGCAACGATGTTACGCAGCAGGGCAGTCGCCCTAAAACAAAGTTAAACATCATGGAGAAAAAAATCACTGGATATACCACC<br>GTTGATATATCCCAATGGCATCGTAAAGAACATTTTGAGGCATTTCAGTCAGTTGCTCAATGTACCTATAACCAGACCGTTCAGCTGGATATTACGGCCTTTTTAAAGACCGTAAAGA<br>AAAATAAGCACAAGTTTTATCCGGCCTTTATTCACATTCTTGCCCGCCTGATGAATGCTCATCCGGAGTTCCGTATGGCAATGAAAGACGGTGAGCTGGTGATATGGGATAGTGTTC<br>ACCCTTGTTACACCGTTTTCCATGAGCAAACTGAAACGTTTTCATCGCTCTGGAGTGAATACCACGACGATTTCCGGCAGTTTCTACACATATATTCGCAAGATGTGGCGTGTTACG<br>GTGAAAACCTGGCCTATTTCCCTAAAGGGTTTATTGAGAATATGTTTTTCGTCTCAGCCAATCCCTGGGTGAGTTTCACCAGTTTTGATTTAAACGTGGCCAATATGGACAACTTCTT<br>CGCCCCCGTTTTCACAATGGGCAAATATTATACGCAAGGCGACAAGGTGCTGATGCCGCTGGCGATTCAGGTTCATCATGCCGTTTGTGATGGCTTCCATGTCGGCAGAATGCTTAA<br>TGAATTACAACAGTACTGCGATGAGTGGCAGGGCGGGGCGTAATTTTTTTAAGGCAGTTATTGGTGCGATCCTCTAGCTAGAAATTCGTTCAAGCCGACGCCGCTTCGCCGGCGTT<br>AACTCAAGCGATTAGATGCACTAAGCACATAATTGCTCACAGCCAAACTATCAGGTCAAGTCTGCTTTTATTATTTTTAAGCGTGCATAATAAGCCCTACGCGTATGACAGTCTCCGG<br>AAGACGGTTGTTGCGCACGTATTCGGTGAACGCACTATGGCGACGCTGGGGCGTCTTATGAGCCTGCTGTCACCCTTTGACGTGGTGATATGGATGACGGATGGCTGGCCGCTGTA<br>TGAATCCCGCCTGAAGGGAAAGCTGCACGTAATCAGCAAGCGATATACGCAGCGAATTGAGCGGCATAACCTGAATCTGAGGCAGCACCTGGCACGGCTGGGACGGAAGTCGCT<br>GTCGTTCTCAAAATCGGTGGAGCTGCATGACAAAGTCATCGGGCATTATCTGAACATAAAACACTATCAATAAGTTGGAGTCATTACCCAATTATGATAGAATTTACAAGCTATAAG<br>GTTATTGTCCTGGGTTTCAAGCATTAGTCCATGCAAGTTTTTATGCTTTGCCCATTCTATAGATATATTGATAAGCGCGCTGCCTATGCCTTGCCCCCTGAAATCCTTACATACGGCGAT<br>ATCTTCTATATAAAAGATATATTATCTTATCAGTATTGTCAATATATTCAAGGCAATCTGCCTCCTCATCCTCTTCATCCTCTTCGTCTTGGTAGCTTTTTAAATATGGCGCTTCATAGAG<br>TAATTCTGTAAAGGTCCAATTCTCGTTTTCATACCTCGGTATAATCTTACCTATCACCTCAAATGGTTCGCTGGGTTTATCGCACCCCCGAACACGAGCACGGCACCCGCGACCACTA<br>TGCCAAGAATGCCCAAGGTAAAAATTGCCGGCCCCGCCATGAAGTCCGTGAATGCCCCGACGGCCGAAGTGAAGGGCAGGCCGCCACCCAGGCCGCCGCCCTCACTGCCCGGC<br>ACCTGGTCGCTGAATGTCGATGCCAGCACCTGCGGCACGTCAATGCTTCCGGGCGTCGCGCTCGGGCTGATCGCCCATCCCGTTACTGCCCCGATCCCGGCAATGGCAAGGACTG<br>CCAGCGCTGCCATTTTTGGGGTGAGGCCGTTCGCGGCCGAGGGGCGCAGCCCCTGGGGGGATGGGAGGCCCGCGTTAGCGGGCCGGGAGGGTTCGAGAAGGGGGGGCACCCCC<br>CTTCGGCGTGCGCGGTCACGCGCACAGGGCGCAGCCCTGGTTAAAAACAAGGTTTATAAATATTGGTTTAAAAGCAGGTTAAAAGACAGGTTAGCGGTGGCCGAAAAACGGGCG<br>GAAACCCTTGCAAATGCTGGATTTTCTGCCTGTGGACAGCCCCTCAAATGTCAATAGGTGCGCCCCTCATCTGTCAGCACTCTGCCCCTCAAGTGTCAAGGATCGCGCCCCTCATC<br>TGTCAGTAGTCGCGCCCCTCAAGTGTCAATACCGCAGGGCACTTATCCCCAGGCTTGTCCACATCATCTGTGGGAAACTCGCGTAAAATCAGGCGTTTTCGCCGATTTGCGAGGCT<br>GGCCAGCTCCACGTCGCCGGCCGAAATCGAGCCTGCCCCTCATCTGTCAACGCCGCGCCGGGTGAGTCGGCCCCTCAAGTGTCAACGTCCGCCCCTCATCTGTCAGTGAGGGCC<br>AAGTTTTCCGCGAGGTATCCACAACGCCGGCGGCCGCGGTGTCTCGCACACGGCTTCGACGGCGTTTCTGGCGCGTTTGCAGGGCCATAGACGGCCGCCAGCCCAGCGGCGAGG<br>GCAACCAGCCCGG

Supplementary Figure S2: pBin-*NtMYC2aΔ*-RI vector

<table>
<tr><td>Vector map</td><td>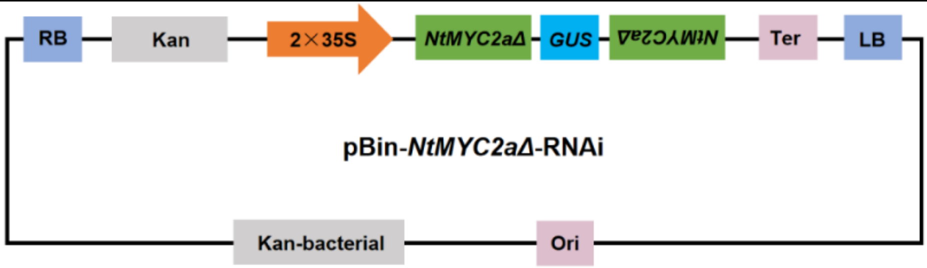
</td></tr>
<tr><td>sector sequence</td><td>TGAGCGTCGCAAAGGCGCTCGGTCTTGCCTTGCTCGTCGGTGATGTACTTCACCAGCTCCGCGAAGTCGCTCTTCTTGATGGAGCGCATGGGGACGTGCTTGGCAATCACGCGC
ACCCCCCGGCCGTTTTAGCGGCTAAAAAAGTCATGGCTCTGCCCTCGGGCGGACCACGCCCATCATGACCTTGCCAAGCTCGTCCTGCTTCTCTTCGATCTTCGCCAGCAGGGC
GAGGATCGTGGCATCACCGAACCGCGCCGTGCGCGGGTCGTCGGTGAGCCAGAGTTTCAGCAGGCCGCCCAGGCGGCCCAGGTCGCCATTGATGCGGGCCAGCTCGCGGACG
TGCTCATAGTCCACGACGCCCGTGATTTTGTAGCCCTGGCCGACGGCCAGCAGGTAGGCCGACAGGCTCATGCCGGCCGCCGCCGCCTTTTCCTCAATCGCTCTTCGTTCGTCTG
GAAGGCAGTACACCTTGATAGGTGGGCTGCCCTTCCTGGTTGGCTTGGTTTCATCAGCCATCCGCTTGCCCTCATCTGTTACGCCGGCGGTAGCCGGCCAGCCTCGCAGAGCAG
GATTCCCGTTGAGCACCGCCAGGTGCGAATAAGGGACAGTGAAGAAGGAACACCCGCTCGCGGGTGGGCCTACTTCACCTATCCTGCCCGGCTGACGCCGTTGGATACACCAA
GGAAAGTCTACACGAACCCTTTGGCAAAATCCTGTATATCGTGCGAAAAAGGATGGATATACCGAAAAAATCGCTATAATGACCCCGAAGCAGGGTTATGCAGCGGAAAAGCGC
CACGCTTCCCGAAGGGAGAAAGGCGGACAGGTATCCGGTAAGCGGCAGGGTCGGAACAGGAGAGCGCACGAGGGAGCTTCCAGGGGGAAACGCCTGGTATCTTTATAGTCCT
GTCGGGTTTCGCCACCTCTGACTTGAGCGTCGATTTTTGTGATGCTCGTCAGGGGGGCGGAGCCTATGGAAAAACGCCAGCAACGCGGCCTTTTTACGGTTCCTGGCCTTTTGCT
GGCCTTTTGCTCACATGTTCTTTCCTGCGTTATCCCCTGATTCTGTGGATAACCGTATTACCGCCTTTGAGTGAGCTGATACCGCTCGCCGCAGCCGAACGACCGAGCGCAGCGAG
TCAGTGAGCGAGGAAGCGGAAGAGCGCCAGAAGGCCGCCAGAGAGGCCGAGCGCGGCCGTGAGGCTTGGACGCTAGGGCAGGGCATGAAAAAGCCCGTAGCGGGCTGCTAC
GGGCGTCTGACGCGGTGGAAAGGGGGAGGGGATGTTGTCTACATGGCTCTGCTGTAGTGAGTGGGTTGCGCTCCGGCAGCGGTCCTGATCAATCGTCACCCTTTCTCGGTCCTT
CAACGTTCCTGACAACGAGCCTCCTTTTCGCCAATCCATCGACAATCACCGCGAGTCCCTGCTCGAACGCTGCGTCCGGACCGGCTTCGTCGAAGGCGTCTATCGCGGCCCGCA
ACAGCGGCGAGAGCGGAGCCTGTTCAACGGTGCCGCCGCGCTCGCCGGCATCGCTGTCGCCGGCCTGCTCCTCAAGCACGGCCCCAACAGTGAAGTAGCTGATTGTCATCAGC
GCATTGACGGCGTCCCCGGCCGAAAAACCCGCCTCGCAGAGGAAGCGAAGCTGCGCGTCGGCCGTTTCCATCTGCGGTGCGCCCGGTCGCGTGCCGGCATGGATGCGCGCGCC
ATCGCGGTAGGCGAGCAGCGCCTGCCTGAAGCTGCGGGCATTCCCGATCAGAAATGAGCGCCAGTCGTCGTCGGCTCTCGGCACCGAATGCGTATGATTCTCCGCCAGCATGGC
TTCGGCCAGTGCGTCGAGCAGCGCCCGCTTGTTCCTGAAGTGCCAGTAAAGCGCCGGCTGCTGAACCCCCAACCGTTCCGCCAGTTTGCGTGTCGTCAGACCGTCTACGCCGA
CCTCGTTCAACAGGTCCAGGGCGGCACGGATCACTGTATTCGGCTGCAACTTTGTCATGCTTGACACTTTATCACTGATAAACATAATATGTCCACCAACTTATCAGTGATAAAGA
ATCCGCGCGTTCAATCGGACCAGCGGAGGCTGGTCCGGAGGCCAGACGTGAAACCCAACATACCCCTGATCGTAATTCTGAGCACTGTCGCGCTCGACGCTGTCGGCATCGGCC
TGATTATGCCGGTGCTGCCGGGCCTCCTGCGCGATCTGGTTCACTCGAACGACGTCACCGCCCACTATGGCATTCTGCTGGCGCTGTATGCGTTGGTGCAATTTGCCTGCGCACCT
GTGCTGGGCGCGCTGTCGGATCGTTTCGGGCGGCGGCCAATCTTGCTCGTCTCGCTGGCCGGCGCCAGATCTGGGGAACCCTGTGGTTGGCATGCACATACAAATGGACGAACG
GATAAACCTTTTCACGCCCTTTTAAATATCCGATTATTCTAATAAACGCTCTTTTCTCTTAGGTTTACCCGCCAATATATCCTGTCAAACACTGATAGTTTAAACTGAAGGCGGGAA
ACGACAATCTGATCATGAGCGGAGAATTAAGGGAGTCACGTTATGACCCCCGCCGATGACGCGGGACAAGCCGTTTTACGTTTGGAACTGACAGAACCGCAACGTTGAAGGAG
CCACTCAGCCGCGGGTTTCTGGAGTTTAATGAGCTAAGCACATACGTCAGAAACCATTATTGCGCGTTCAAAAGTCGCCTAAGGTCACTATCAGCTAGCAAATATTTCTTGTCAA
AAATGCTCCACTGACGTTCCATAAATTCCCCTCGGTATCCAATTAGAGTCTCATATTCACTCTCAATCCAAATAATCTGCACCGGATCTGGATCGTTTCGCATGATTGAACAAGATG
GATTGCACGCAGGTTCTCCGGCCGCTTGGGTGGAGAGGCTATTCGGCTATGACTGGGCACAACAGACAATCGGCTGCTCTGATGCCGCCGTGTTCCGGCTGTCAGCGCAGGGGC
GCCCGGTTCTTTTTGTCAAGACCGACCTGTCCGGTGCCCTGAATGAACTGCAGGACGAGGCAGCGCGGCTATCGTGGCTGGCCACGACGGGCGTTCCTTGCGCAGCTGTGCTC
GACGTTGTCACTGAAGCGGGAAGGGACTGGCTGCTATTGGGCGAAGTGCCGGGGCAGGATCTCCTGTCATCTCACCTTGCTCCTGCCGAGAAAGTATCCATCATGGCTGATGCA
ATGCGGCGGCTGCATACGCTTGATCCGGCTACCTGCCCATTCGACCACCAAGCGAAACATCGCATCGAGCGAGCACGTACTCGGATGGAAGCCGGTCTTGTCGATCAGGATGAT
CTGGACGAAGAGCATCAGGGGCTCGCGCCAGCCGAACTGTTCGCCAGGCTCAAGGCGCGCATGCCCGACGGCGATGATCTCGTCGTGACCCATGGCGATGCCTGCTTGCCGAA
TATCATGGTGGAAAATGGCCGCTTTTCTGGATTCATCGACTGTGGCCGGCTGGGTGTGGCGGACCGCTATCAGGACATAGCGTTGGCTACCCGTGATATTGCTGAAGAGCTTGGC
GGCGAATGGGCTGACCGCTTCCTCGTGCTTTACGGTATCGCCGCTCCCGATTCGCAGCGCATCGCCTTCTATCGCCTTCTTGACGAGTTCTTCTGAGCGGGACTCTGGGGTTCGA
AATGACCGACCAAGCGACGCCCAACCTGCCATCACGAGATTTCGATTCCACCGCCGCCTTCTATGAAAGGTTGGGCTTCGGAATCGTTTTCCGGGACGCCGGCTGGATGATCCTC
CAGCGCGGGGATCTCATGCTGGAGTTCTTCGCCCACGGGATCTCTGCGGAACAGGCGGTCGAAGGTGCCGATATCATTACGACAGCAACGGCCGACAAGCACAACGCCACGAT
CCTGAGCGACAATATGATCGGGCCCGGCGTCCACATCAACGGCGTCGGCGGCGACTGCCCAGGCAAGACCGAGATGCACCGCGATATCTTGCTGCGTTCGGATATTTTCGTGGA
GTTCCCGCCACAGACCCGGATGATCCCCGATCGTTCAAACATTTGGCAATAAAGTTTCTTAAGATTGAATCCTGTTGCCGGTCTTGCGATGATTATCATATAATTTCTGTTGAATTA
CGTTAAGCATGTAATAATTAACATGTAATGCATGACGTTATTTATGAGATGGGTTTTTATGATTAGAGTCCCGCAATTATACATTTAATACGCGATAGAAAACAAAATATAGCGCGCA
AACTAGGATAAATTATCGCGCGCGGTGTCATCTATGTTACTAGATCGGGCCTCCTGTCAATGCTGGCGGCGGCTCTGGTGGTGGTTCTGGTGGCGGCTCTGAGGGTGGTGGCTCT
GAGGGTGGCGGTTCTGAGGGTGGCGGCTCTGAGGGAGGCGGTTCCGGTGGTGGCTCTGGTTCCGGTGATTTTGATTATGAAAAGATGGCAAACGCTAATAAGGGGGCTATGACC
GAAAATGCCGATGAAAACGCGCTACAGTCTGACGCTAAAGGCAAACTTGATTCTGTCGCTACTGATTACGGTGCTGCTATCGATGGTTTCATTGGTGACGTTTCCGGCCTTGCTA
ATGGTAATGGTGCTACTGGTGATTTTGCTGGCTCTAATTCCCAAATGGCTCAAGTCGGTGACGGTGATAATTCACCTTTAATGAATAATTTCCGTCAATATTTACCTTCCCTCCCTCA
ATCGGTTGAATGTCGCCCTTTTGTCTTTGGCCCAATACGCAAACCGCCTCTCCCCGCGCGTTGGCCGATTCATTAATGCAGCTGGCACGACAGGTTTCCCGACTGGAAAGCGGGC
AGTGAGCGCAACGCAATTAATGTGAGTTAGCTCACTCATTAGGCACCCCAGGCTTTACACTTTATGCTTCCGGCTCGTATGTTGTGTGGAATTGTGAGCGGATAACAATTTCACAC
AGGAAACAGCTATGACCATGATTACGCCAAGCTTGATATCGAATTCCTGCAGGTCAACATGGTGGAGCACGACACACTTGTCTACTCCAAAAATATCAAAGATACAGTCTCAGAA
GACCAAAGGGCAATTGAGACTTTTCAACAAAGGGTAATATCCGGAAACCTCCTCGGATTCCATTGCCCAGCTATCTGTCACTTTATTGTGAAGATAGTGGAAAAGGAAGGTGGCT
CCTACAAATGCCATCATTGCGATAAAGGAAAGGCCATCGTTGAAGATGCCTCTGCCGACAGTGGTCCCAAAGATGGACCCCCACCCACGAGGAGCATCGTGGAAAAAGAAGAC
GTTCCAACCACGTCTTCAAAGCAAGTGGATTGATGTGATAACATGGTGGAGCACGACACACTTGTCTACTCCAAAAATATCAAAGATACAGTCTCAGAAGACCAAAGGGCAATT
GAGACTTTTCAACAAAGGGTAATATCCGGAAACCTCCTCGGATTCCATTGCCCAGCTATCTGTCACTTTATTGTGAAGATAGTGGAAAAGGAAGGTGGCTCCTACAAATGCCATC
ATTGCGATAAAGGAAAGGCCATCGTTGAAGATGCCTCTGCCGACAGTGGTCCCAAAGATGGACCCCCACCCACGAGGAGCATCGTGGAAAAAGAAGACGTTCCAACCACGTCT
TCAAAGCAAGTGGATTGATGTGATATCTCCACTGACGTAAGGGATGACGCACAATCCCACTATCCTTCGCAAGACCCTTCCTCTATATAAGGAAGTTCATTTCATTTGGAGAGGAC
CTCGAGAATTCTCAACACAACATATACAAAACAAACGAATCTCAAGCAATCAAGCATTCTACTTCTATTGCAGCAATTTAAATCATTTCTTTTAAAGCAAAAGCAATTTTCTGAAA
ATTTTCACCATTTACGAACGATAGGCTCGAATTATCACAAGTTTGTACAAAAAAGCATGACTGATTACAGCTTACCCACCATGAATTTGTGGAATACTAGTGGTACTACCGATGAC
AACGTTTCTATGATGGAATCTTTTATGTCTTCTGATCTCACTTCATTTTGGGCTACTTCTAATTCTACTACTGCTGCTGTTACCTCTAATTCTAATCTTATTCCAGTTAATACCCTAACT
GTTCTTCTTCCGTCTTCTTGTGCTTCTACTGTCACAGCTGTGGCTGTCGATGCTTCAAAATCCATGTCTTTTTCAACCAAGAAACTCTTCAGCAGCGTCTTCAAACCCTCATTGAT
GGTGCTCGTGAGACGTGGACCTATGCCATCTTTTGGCAGTCATCCGTCGTTGATTTATCGAGTCCGTTTGTGTTGGGCTGGGGAGATGGTTACTACAAAGGTGAAGAAGATAAAG
CCAATAGGAAATTAGCTGTTTCTTCTCCTGCTTATATTGCTGAGCAAGAACACCGAAAAAAGGTTCTCCGGGAGCTGAATTCGTTGATCTCCGGCACGCAAACCGGCACTGATGA
TGCCGTCGATGAAGAAGTTACCGACACTGAATGGTTCTTCCTTATTTCCATGACCCAATCGTTTGTTAACGGAAGTGGGCTTCCCGCGCCGACCCAGCTTTCTTGTACAAAGTGG
TGATAGCTTGGCGCGCCAGAGATAACCTTCACCCGGTTGCCAGAGGTGCGGATTCACCACTTGCAAAGTCCCGCTAGTGCCTTGTCCAGTTGCAACCACCTGTTGATCCGCATCA
CGCAGTTCAACGCTGACATCACCATTGGCCACCACCTGCCAGTCAACAGACGCGTGGTTACAGTCTTGCGCGACATGCGTCACCACGGTGATATCGTCCACCCAGGTGTTCGGC
GTGGTGTAGAGCATTACGCTGCGATGGATTCCGGCATAGTTAAAGAAATCATGGAAGTAAGACTGCTTTTTCTTGCCGTTTTCGTCGGTAATCACCATTCCCGGCGGGATAGTCTG
CCAGTTCAGTTCGTTGTTCACACAAACGGTGATACGTACACTTTTCCCGGCAATAACATACGGCGTGACATCTTAATTAAGAATTATCGAACCACTTTGTACAAGAAAGCTGGGT
CGGCGCGGAAGCCCACTTCCGTTAACAAACGATTGGGTCATGGAAATAAGGAAGAACCATTCAGTGTCGGTAACTTCTTCATCGACGGCATCATCAGTGCCGGTTTGCGTGCCG
GAGATCAACGAATTCAGCTCCCGGAGAACCTTTTTTCGGTGTTCTTGCTCAGCAATATAAGCAGGAGAAGAAACAGCTAATTTCCTATTGGCTTTATCTTCTTCACCTTTGTAGTA
ACCATCTCCCCAGCCCAACACAAACGGACTCGATAAATCAACGACGGATGACTGCCAAAAGATGGCATAGGTCCACGTCTCACGAGCACCATCAATGAGGGTTTGAAGACGCT
GCTGAAGAGTTTCTTGGTTGAAAAAAGACATGGATTTTGAAGCATCGACAGCCACAGCTGTGACAGTAGAAGCACAAGAAGACGGAAGAAGAACAGTTAGGGTATTAACTGG
AATAAGATTAGAATTAGAGGTAACAGCAGCAGTAGTAGAATTAGAAGTAGCCCAAAATGAAGTGAGATCAGAAGACATAAAAGATTCCATCATAGAAACGTTGTCATCGGTAGT
ACCACTAGTATTCCACAAATTCATGGTGGGTAAGCTGTAATCAGTCATCCGCGGAGCCTGCTTTTTTGTACAAACTTGTCCTAGTTCTAGAGTCCGCAAAAATCACCAGTCTCTCT
CTACAAATCTATCTCTCTCTATTTTTCTCCAGAATAATGTGTGAGTAGTTCCCAGATAAGGGAATTAGGGTTCTTATAGGGTTTCGCTCATGTGTTGAGCATATAAGAAACCCTTAGT
ATGTATTTGTATTTGTAAAATACTTCTATCAATAAAATTTCTAATTCCTAAAACCAAAATCCAGTGACCTGCAGCCCGGGGGATCCACTAGAGCGGCCGCCACCGCGGTGGAGGGG
GGATCCACTAGAGCGGCCGCCACCGGGTACCGAGCTCGAATTCACTGGCCGTCGTTTTACAACGTCGTGACTGGGAAAACCCTGGCGTTACCCAACTTAATCGCCTTGCAGCAC
ATCCCCCTTTCGCCAGCTGGCGTAATAGCGAAGAGGCCCGCACCGATCGCCCTTCCCAACAGTTGCGCAGCCTGAATGGCGCCCGCTCCTTTCGCTTTCTTCCCTTCCTTTCTCG
CCACGTTCGCCGGCTTTCCCCGTCAAGCTCTAAATCGGGGGCTCCCTTTAGGGTTCCGATTTAGTGCTTTACGGCACCTCGACCCCAAAAAACTTGATTTGGGTGATGGTTCACG
TAGTGGGCCATCGCCCTGATAGACGGTTTTTCGCCCTTTGACGTTGGAGTCCACGTTCTTTAATAGTGGACTCTTGTTCCAAACTGGAACAACACTCAACCCTATCTCGGGCTATT
CTTTTGATTTATAAGGGATTTTGCCGATTTCGGAACCACCATCAAACAGGATTTTCGCCTGCTGGGGCAAACCAGCGTGGACCGCTTGCTGCAACTCTCTCAGGGCCAGGCGGTG
AAGGGCAATCAGCTGTTGCCCGTCTCACTGGTGAAAAGAAAAACCACCCCAGTACATTAAAAACGTCCGCAATGTGTTATTAAGTTGTCTAAGCGTCAATTTGTTTACACCACAA
TATATCCTGCCACCAGCCAGCCAACAGCTCCCCGACCGGCAGCTCGGCACAAAATCACCACTCGATACAGGCAGCCCATCAGTCCGGGACGGCGTCAGCGGGAGAGCCGTTGT
AAGGCGGCAGACTTTGCTCATGTTACCGATGCTATTCGGAAGAACGGCAACTAAGCTGCCGGGTTTGAAACACGGATGATCTCGCGGAGGGTAGCATGTTGATTGTAACGATGA
CAGAGCGTTGCTGCCTGTGATCAAATATCATCTCCCTCGCAGAGATCCGAATTATCAGCCTTCTTATTCATTTCTCGCTTAACCGTGACAGGCTGTCGATCTTGAGAACTATGCCGA
CATAATAGGAAATCGCTGGATAAAGCCGCTGAGGAAGCTGAGTGGCGCTATTTCTTTAGAAGTGAACGTTGACGATATCAACTCCCCTATCCATTGCTCACCGAATGGTACAGGT
CGGGGACCCGAAGTTCCGACTGTCGGCCTGATGCATCCCCGGCTGATCGACCCCAGATCTGGGGCTGAGAAAGCCCAGTAAGGAAACAACTGTAGGTTCGAGTCGCGAGATCC
CCCGGAACCAAAGGAAGTAGGTTAAACCCGCTCCGATCAGGCCGAGCCACGCCAGGCCGAGAACATTGGTTCCTGTAGGCATCGGGATTGGCGGATCAAACACTAAAGCTACT
GGAACGAGCAGAAGTCCTCCGGCCGCCAGTTGCCAGGCGGTAAAGGTGAGCAGAGGCACGGGAGGTTGCCACTTGCGGGTCAGCACGGTTCCGAACGCCATGGAAACCGCC
CCCGCCAGGCCCGCTGCGACGCCGACAGGATCTAGCGCTGCGTTTGGTGTCAACACCAACAGCGCCACGCCCGCAGTTCCGCAAATAGCCCCCAGGACCGCCATCAATCGTATC
GGGCTACCTAGCAGAGCGGCAGAGATGAACACGACCATCAGCGGCTGCACAGCGCCTACCGTCGCCGCGACCCCGCCCGGCAGGCGGTAGACCGAAATAAACAACAAGCTCC
AGAATAGCGAAATATTAAGTGCGCCGAGGATGAAGATGCGCATCCACCAGATTCCCGTTGGAATCTGTCGGACGATCATCACGAGCAATAAACCCGCCGGCAACGCCCGCAGCA
GCATACCGGCGACCCCTCGGCCTCGCTGTTCGGGCTCCACGAAAACGCCGGACAGATGCGCCTTGTGAGCGTCCTTGGGGCCGTCCTCCTGTTTGAAGACCGACAGCCCAATGA
TCTCGCCGTCGATGTAGGCGCCGAATGCCACGGCATCTCGCAACCGTTCAGCGAACGCCTCCATGGGCTTTTTCTCCTCGTGCTCGTAAACGGACCCGAACATCTCTGGAGCTTT
CTTCAGGGCCGACAATCGGATCTCGCGGAAATCCTGCACGTCGGCCGCTCCAAGCCGTCGAATCTGAGCCTTAATCACAATTGTCAATTTTAATCCTCTGTTTATCGGCAGTTCGT</td></tr>
</table>

	AGAGCGCGCCGTGCGTCCCGAGCGATACTGAGCGAAGCAAGTGCGTCGAGCAGTGCCCGCTTGTTCCTGAAATGCCAGTAAAGCGCTGGCTGCTGAACCCCCAGCCGGAACT<br>GACCCCACAAGGCCCTAGCGTTTGCAATGCACCAGGTCATCATTGACCCAGGCGTGTTCCACCAGGCCGCTGCCTCGCAACTCTTCGCAGGCTTCGCCGACCTGCTCGCGCCAC<br>TTCTTCACGCGGGTGGAATCCGATCCGCACATGAGGCGGAAGGTTTCCAGCTTGAGCGGGTACGGCTCCCGGTGCGAGCTGAAATAGTCGAACATCCGTCGGGCCGTCGGCGA<br>CAGCTTGCGGTACTTCTCCCATATGAATTTCGTGTAGTGGTCGCCAGCAAACAGCACGACGATTTCCTCGTCGATCAGGACCTGGCAACGGGACGTTTTCTTGCCACGGTCCAGG<br>ACGCGGAAGCGGTGCAGCAGCGACACCGATTCCAGGTGCCCAACGCGGTCGGACGTGAAGCCCATCGCCGTCGCCTGTAGGCGCGACAGGCATTCCTCGGCCTTCGTGTAATA<br>CCGGCCATTGATCGACCAGCCCAGGTCCTGGCAAAGCTCGTAGAACGTGAAGGTGATCGGCTCGCCGATAGGGGTGCGCTTCGCGTACTCCAACACCTGCTGCCACACCAGTTC<br>GTCATCGTCGGCCCGCAGCTCGACGCCGGTGTAGGTGATCTTCACGTCCTTGTTGACGTGGAAAATGACCTTGTTTTGCAGCGCCTCGCGCGGGATTTTCTTGTTGCGCGTGGTG<br>AACAGGGCAGAGCGGGCCGTGTCGTTTGGCATCGCTCGCATCGTGTCCGGCCACGGCGCAATATCGAACAAGGAAAGCTGCATTTCCTTGATCTGCTGCTTCGTGTGTTTCAGC<br>AACGCGGCCTGCTTGGCCTCGCTGACCTGTTTTGCCAGGTCCTCGCCGGCGGTTTTTCGCTTCTTGGTCGTCATAGTTCCTCGCGTGTCGATGGTCATCGACTTCGCCAAACCTG<br>CCGCCTCCTGTTCGAGACGACGCGAACGCTCCACGGCGGCCGATGGCGCGGGCAGGGCAGGGGGAGCCAGTTGCACGCTGTCGCGCTCGATCTTGGCCGTAGCTTGCTGGACC<br>ATCGAGCCGACGGACTGGAAGGTTTCGCGGGGCGCACGCATGACGGTGCGGCTTGCGATGGTTTCGGCATCCTCGGCGGAAAACCCCGCGTCGATCAGTTCTTGCCTGTATGCC<br>TTCCGGTCAAACGTCCGATTCATTCACCCTCCTTGCGGGATTGCCCCGACTCACGCCGGGGCAATGTGCCCTTATTCCTGATTTGACCCGCCTGGTGCCTTGGTGTCCAGATAATC<br>CACCTTATCGGCAATGAAGTCGGTCCCGTAGACCGTCTGGCCGTCCTTCTCGTACTTGGTATTCCGAATCTTGCCCTGCACGAATACCAGCGACCCCTTGCCCAAATACTTGCCGT<br>GGGCCTCGGCCTGAGAGCCAAAACACTTGATGCGGAAGAAGTCGGTGCGCTCCTGCTTGTCGCCGGCATCGTTGCGCCACATCTAGGTACTAAAACAATTCATCCAGTAAAATA<br>TAATATTTTATTTTCTCCCAATCAGGCTTGATCCCCAGTAAGTCAAAAAATAGCTCGACATACTGTTCTTCCCCGATATCCTCCCTGATCGACCGGACGCAGAAGGCAATGTCATAC<br>CACTTGTCCGCCCTGCCGCTTCTCCCAAGATCAATAAAGCCACTTACTTTGCCATCTTTCACAAAGATGTTGCTGTCTCCCAGGTCGCCGTGGGAAAAGACAAGTTCCTCTTCGG<br>GCTTTTCCGTCTTTAAAAAATCATACAGCTCGCGCGGATCTTTAAATGGAGTGTCTTCTTCCCAGTTTTCGCAATCCACATCGGCCAGATCGTTATTCAGTAAGTAATCCAATTCGG<br>CTAAGCGGCTGTCTAAGCTATTCGTATAGGGACAATCCGATATGTCGATGGAGTGAAAGAGCCTGATGCACTCCGCATACAGCTCGATAATCTTTTCAGGGCTTTGTTCATCTTCAT<br>ACTCTTCCGAGCAAAGGACGCCATCGGCCTCACTCATGAGCAGATTGCTCCAGCCATCATGCCGTTCAAAGTGCAGGACCTTTGGAACAGGCAGCTTTCCTTCCAGCCATAGCA<br>TCATGTCCTTTTCCCGTTCCACATCATAGGTGGTCCCTTTATACCGGCTGTCCGTCATTTTTAAATATAGGTTTTCATTTTCTCCCACCAGCTTATATACCTTAGCAGGAGACATTCCT<br>TCCGTATCTTTTACGCAGCGGTATTTTTCGATCAGTTTTTTCAATTCCGGTGATATTCTCATTTTAGCCATTTATTATTTCCTTCCTCTTTTCTACAGTATTTAAAGATACCCCAAGAAG<br>CTAATTATAACAAGACGAACTCCAATTCACTGTTCCTTGCATTCTAAAACCTTAAATACCAGAAAACAGCTTTTTCAAAGTTGTTTTCAAAGTTGGCGTATAACATAGTATCGACG<br>GAGCCGATTTTGAAACCACAATTATGGGTGATGCTGCCAACTTACTGATTTAGTGTATGATGGTGTTTTTGAGGTGCTCCAGTGGCTTCTGTGTCTATCAGCTGTCCCTCCTGTTCA<br>GCTACTGACGGGGTGGTGCGTAACGGCAAAAGCACCGCCGGACATCAGCGCTATCTCTGCTCTCACTGCCGTAAAACATGGCAACTGCAGTTCACTTACACCGCTTCTCAACCC<br>GGTACGCACCAGAAAATCATTGATATGGCCATGAATGGCGTTGGATGCCGGGCAACAGCCCGCATTATGGGCGTTGGCCTCAACACGATTTTACGTCACTTAAAAAACTCAGGCC<br>GCAGTCGGTAACCTCGCGCATACAGCCGGGCAGTGACGTCATCGTCTGCGCGGAAATGGACGAACAGTGGGGCTATGTCGGGGCTAAATCGCGCCAGCGCTGGCTGTTTTACGC<br>GTTGGGAGCTCTCCCATATGGTCGACCTGCAGGCGGCCGCGAATTCACTAGTGATACCCCATATGTGATCAATTCGGGCACGAACCCAGTGGACATAAGCCTCGTTCGGTTCGTA<br>AGCTGTAATGCAAGTAGCGTAACTGCCGTCACGCAACTGGTCCAGAACCTTGACCGAACGCAGCGGTGGTAACGGCGCAGTGGCGGTTTTCATGGCTTCTTGTTATGACATGTT<br>TTTTTGGGGTACAGTCTATGCCTCGGGCATCCAAGCAGCAAGCGCGTTACGCCGTGGGTCGATGTTTGATGTTATGGAGCAGCAACGATGTTACGCAGCAGGGCAGTCGCCCTA<br>AAACAAAGTTAAACATCATGGAGAAAAAAATCACTGGATATACCACCGTTGATATATCCCAATGGCATCGTAAAGAACATTTTGAGGCATTTCAGTCAGTTGCTCAATGTACCTAT<br>AACCAGACCGTTCAGCTGGATATTACGGCCTTTTTAAAGACCGTAAAGAAAAATAAGCACAAGTTTTATCCGGCCTTTATTCACATTCTTGCCCGCCTGATGAATGCTCATCCGGA<br>GTTCCGTATGGCAATGAAAGACGGTGAGCTGGTGATATGGGATAGTGTTCACCCTTGTTACACCGTTTTCCATGAGCAAACTGAAACGTTTTCATCGCTCTGGAGTGAATACCAC<br>GACGATTTCCGGCAGTTTCTACACATATATTCGCAAGATGTGGCGTGTTACGGTGAAAACCTGGCCTATTTCCCTAAAGGGTTTATTGAGAATATGTTTTTCGTCTCAGCCAATCCC<br>TGGGTGAGTTTCACCAGTTTTGATTTAAACGTGGCCAATATGGACAACTTCTTCGCCCCCGTTTTCACAATGGGCAAATATTATACGCAAGGCGACAAGGTGCTGATGCCGCTGG<br>CGATTCAGGTTCATCATGCCGTTTGTGATGGCTTCCATGTCGGCAGAATGCTTAATGAATTACAACAGTACTGCGATGAGTGGCAGGGCGGGGCGTAATTTTTTTAAGGCAGTTAT<br>TGGTGCGATCCTCTAGCTAGAAATTCGTTCAAGCCGACGCCGCTTCGCCGGCGTTAACTCAAGCGATTAGATGCACTAAGCACATAATTGCTCACAGCCAAACTATCAGGTCAAG<br>TCTGCTTTTATTATTTTTAAGCGTGCATAATAAGCCCTACGCGTATGACAGTCTCCGGAAGACGGTTGTTGCGCACGTATTCGGTGAACGCACTATGGCGACGCTGGGGCGTCTTA<br>TGAGCCTGCTGTCACCCTTTGACGTGGTGATATGGATGACGGATGGCTGGCCGCTGTATGAATCCCGCCTGAAGGGAAAGCTGCACGTAATCAGCAAGCGATATACGCAGCGAAT<br>TGAGCGGCATAACCTGAATCTGAGGCAGCACCTGGCACGGCTGGGACGGAAGTCGCTGTCGTTCTCAAAATCGGTGGAGCTGCATGACAAAGTCATCGGGCATTATCTGAACAT<br>AAAACACTATCAATAAGTTGGAGTCATTACCCAATTATGATAGAATTTACAAGCTATAAGGTTATTGTCCTGGGTTTCAAGCATTAGTCCATGCAAGTTTTTATGCTTTGCCCATTCT<br>ATAGATATATTGATAAGCGCGCTGCCTATGCCTTGCCCCCTGAAATCCTTACATACGGCGATATCTTCTATATAAAAGATATATTATCTTATCAGTATTGTCAATATATTCAAGGCAATC<br>TGCCTCCTCATCCTCTTCATCCTCTTCGTCTTGGTAGCTTTTTAAATATGGCGCTTCATAGAGTAATTCTGTAAAGGTCCAATTCTCGTTTTCATACCTCGGTATAATCTTACCTATCA<br>CCTCAAATGGTTCGCTGGGTTTATCGCACCCCCGAACACGAGCACGGCACCCGCGACCACTATGCCAAGAATGCCCAAGGTAAAAATTGCCGGCCCCGCCATGAAGTCCGTGAA<br>TGCCCCGACGGCCGAAGTGAAGGGCAGGCCGCCACCCAGGCCGCCGCCCTCACTGCCCGGCACCTGGTCGCTGAATGTCGATGCCAGCACCTGCGGCACGTCAATGCTTCCGG<br>GCGTCGCGCTCGGGCTGATCGCCCATCCCGTTACTGCCCCGATCCCGGCAATGGCAAGGACTGCCAGCGCTGCCATTTTTGGGGTGAGGCCGTTCGCGGCCGAGGGGCGCAGCC<br>CCTGGGGGGATGGAGGCCCGCGTTAGCGGGCCGGGAGGGTTCGAGAAGGGGGGGCACCCCCCTTCGGCGTGCGCGGTCACGCGCACAGGGCGCAGCCCTGGTTAAAAACA<br>AGGTTTATAAATATTGGTTTAAAAGCAGGTTAAAAGACAGGTTAGCGGTGGCCGAAAAACGGGCGGAAACCCTTGCAAATGCTGGATTTTCTGCCTGTGGACAGCCCCTCAAAT<br>GTCAATAGGTGCGCCCCTCATCTGTCAGCACTCTGCCCCTCAAGTGTCAAGGATCGCGCCCCTCATCTGTCAGTAGTCGCGCCCCTCAAGTGTCAATACCGCAGGGCACTTATCC<br>CCAGGCTTGTCCACATCATCTGTGGGAAACTCGCGTAAAATCAGGCGTTTTCGCCGATTTGCGAGGCTGGCCAGCTCCACGTCGCCGGCCGAAATCGAGCCTGCCCCTCATCTG<br>TCAACGCCGCGCCGGGTGAGTCGGCCCCTCAAGTGTCAACGTCCGCCCCTCATCTGTCAGTGAGGGCCAAGTTTTCCGCGAGGTATCCACAACGCCGGCGGCCGCGGTGTCTC<br>GCACACGGCTTCGACGGCGTTTCTGGCGCGTTTGCAGGGCCATAGACGGCCGCCAGCCCAGCGGCGAGGGCAACCAGCCCGG
